# The rigid hybrid number for two phylogenetic trees

**DOI:** 10.1007/s00285-021-01594-2

**Published:** 2021-03-26

**Authors:** Katharina T. Huber, Simone Linz, Vincent Moulton

**Affiliations:** 1 School of Computing Sciences, University of East Anglia, Norwich, UK; 2 School of Computer Science, University of Auckland, Auckland, New Zealand

**Keywords:** Phylogenetic network, Hybrid number, Cherry-picking sequence, Fork-picking sequence, Weakly displaying, Rigidly displaying, Temporal tree-child network, Beaded tree, 05C05, 92D15

## Abstract

Recently there has been considerable interest in the problem of finding a phylogenetic network with a minimum number of reticulation vertices which displays a given set of phylogenetic trees, that is, a network with minimum hybrid number. Such networks are useful for representing the evolution of species whose genomes have undergone processes such as lateral gene transfer and recombination that cannot be represented appropriately by a phylogenetic tree. Even so, as was recently pointed out in the literature, insisting that a network displays the set of trees can be an overly restrictive assumption when modeling certain evolutionary phenomena such as incomplete lineage sorting. In this paper, we thus consider the less restrictive notion of rigidly displaying which we introduce and study here. More specifically, we characterize when two trees can be rigidly displayed by a certain type of phylogenetic network called a temporal tree-child network in terms of fork-picking sequences. These are sequences of special subconfigurations of the two trees related to the well-studied cherry-picking sequences. We also show that, in case it exists, the rigid hybrid number for two phylogenetic trees is given by a minimum weight fork-picking sequence for the trees. Finally, we consider the relationship between the rigid hybrid number and three closely related numbers; the weak, beaded, and temporal hybrid numbers. In particular, we show that these numbers can all be different even for a fixed pair of trees, and also present an infinite family of pairs of trees which demonstrates that the difference between the rigid hybrid number and the temporal-hybrid number for two phylogenetic trees on the same set of *n* leaves can grow at least linearly with *n*.

## Introduction

Recently there has been great interest in using phylogenetic networks to model processes such as lateral gene transfer and recombination (see e.g. Bapteste et al. 2013). Such networks come in various forms (see e.g. Huson et al. 2010), and here we shall consider *explicit networks* which are used to provide direct representations of evolutionary histories in the form of a leaf-labelled directed graph. Formally speaking, a *phylogenetic network (on a species set X)* is a connected directed acyclic graph, with a single root and leaf-set *X* in which every internal vertex has either indegree one and outdegree two, or indegree two and outdegree one, except for the root which has outdegree two. We call the number of vertices in a phylogenetic network with indegree two the network’s *hybrid number*, so that a *phylogenetic tree* is a network with hybrid number zero. We shall mainly focus on *temporal tree-child* networks, i.e. phylogenetic networks in which each non-leaf vertex has a child whose indegree is one, whose vertices can be labelled with time stamps that move strictly forward on treelike parts of the network, and so that vertices with indegree two have parents with the same time stamp [also known as tree-child, time-consistent networks (Cardona et al. 2009a)].

Any phylogenetic network on some set *X* displays a set of phylogenetic trees on *X*, where a phylogenetic tree is *displayed* by a network if there is a subgraph of the network that is isomorphic to a subdivision of the tree (van Iersel et al. 2010). Biologically speaking, we usually think of the trees displayed by a network as being gene trees, and the fact that a network is required to represent them simultaneously is the consequence of incongruence between the gene trees that can arise from processes such as lateral gene transfer (Zhu et al. 2016). As gene trees are now commonly inferred from genomic data (e.g. by considering either a gene or a genomic locus of interest) it is therefore natural to try and devise ways to construct phylogenetic networks through the process of looking for some network which displays a given set of gene trees (see e.g. Nakhleh 2010, Section 2). For a given set of phylogenetic trees, this leads to the concept of the *(temporal) hybrid number*, which is the minimum hybrid number taken over all (temporal tree-child) networks that display each of the trees in the set (Baroni et al. 2005b; Humphries et al. 2013a). While the hybrid number exists for any set of phylogenetic trees, it is worth noting that the temporal hybrid number does not always exist, i.e. there are sets of trees that cannot be displayed simultaneously in a temporal tree-child network.

Several results have been presented in the literature concerning displaying phylogenetic trees and hybrid numbers, mainly for pairs of trees. These include structural information on how the hybrid number is related to a so-called *maximum acyclic agreement forest* for two phylogenetic trees (Baroni et al. 2005a), characterizations for when collections of trees are displayed by special types of networks (Humphries et al. 2013a; Linz and Semple 2019) and related algorithms/complexity results (Bordewich and Semple 2007a, b; Döcker et al. 2019; Humphries et al. 2013b; Huson and Linz 2016). However, all of these results rely on the fact that the networks *display* the set of trees in question. Recently it has been observed that this is a serious issue when modelling a phenomenon called *incomplete lineage sorting* where the aim is to model gene tree incongruence arising due to population effects. This is because the set of displayed trees is then no longer able to fully capture the way in which the genes actually evolve along the network, making it difficult to recover the underlying network (Zhu et al. 2016; Zhu and Degnan 2016; Degnan 2016).

**Fig. 1:**
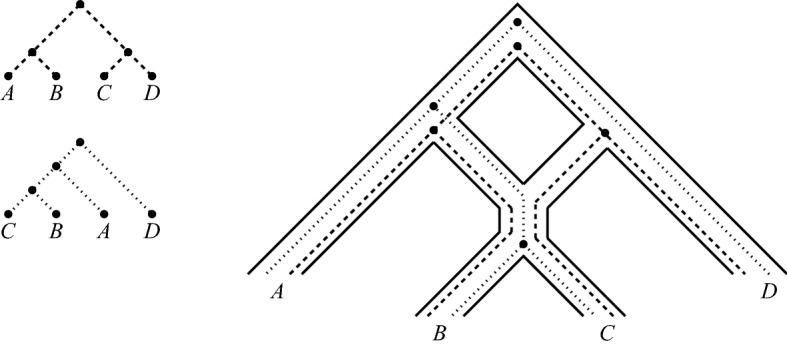
Two phylogenetic trees on the set $\{A,B,C,D\}$ that are rigidly displayed by the phylogenetic network on the right. Note that the tree in the top left is *not* displayed by the network. The trees and network are adapted from (Zhu et al. 2016, Fig. 2, Fig. 3) and represent a hypothetical evolutionary scenario tracing the evolution of a genomic region within four species

A possible solution to this problem is to relax the displaying condition. Roughly speaking, a phylogenetic tree is *weakly displayed* by a network (Huber et al. 2016) if it can be embedded in the network in such a way that the tree follows along the directed paths in the network (see Sect. 3 for the definition). In biological terms, as nicely explained in van Iersel et al. (2018), “this means that different lineages of the gene tree may “travel down” the same branch of the network, as long as any branching node in the tree coincides with a branching node in the network”. In this paper, we focus on the special situation where two phylogenetic trees ${{\mathscr {T}}}$ and ${{\mathscr {T}}}'$ are weakly displayed by a temporal tree-child network under the assumption that there exist simultaneous embeddings of both trees that do not permit more than three branches of ${{\mathscr {T}}}$ and ${{\mathscr {T}}}'$ to come together at a reticulation vertex. In this case we shall say that ${{\mathscr {T}}}$ and ${{\mathscr {T}}}'$ are *rigidly displayed* by the network (see Fig. 1 for an example). As relatively little is known about the problem of constructing phylogenetic networks that weakly display a collection of phylogenetic trees, we believe that our results on rigidly displaying provide some useful new insights into approaching this challenging problem. Ultimately this should hopefully also lead to improved approaches to modeling phenomena such as incomplete lineage sorting.

We now summarize the rest of the paper, including statements of our main results. After presenting some definitions in Sect. 2, in Sect. 3 we present the definition of weakly displaying, and we prove some basic facts concerning this concept that are useful later on. In addition, in Theorem 1 we characterize when two phylogenetic trees are displayed by a temporal tree-child network and when they are weakly displayed such a network.

In Sects. 4 and 5, we introduce the concepts of rigidly displaying and fork-operations, respectively, and prove some results concerning these concepts which we use later on. A fork-operation can be thought of as a generalization of picking off a pair of cherries from two phylogenetic trees as defined in Humphries et al. (2013a). In particular, in a key result, Proposition 1, we show that in case a temporal tree-child network rigidly displays two phylogenetic trees, there exists a certain sequence of fork-operations (called a *special sequence*) which can be applied to these two trees that allows us to apply inductive arguments to prove our main results. Using this, in Theorem 2 we prove that a pair of phylogenetic trees can be rigidly displayed by a temporal tree-child network if and only if there exists a fork-picking sequence for the two trees. Interestingly, we also prove that this is equivalent to the existence of a temporal tree-child network that *displays* the two trees. As a corollary we show that it is NP-complete to decide whether or not there exists a temporal tree-child network that rigidly displays two phylogenetic trees (Corollary 1).

In Sect. 7, we define the rigid hybrid number of two trees, which is the minimum hybrid number of a phylogenetic network that rigidly displays both trees, taken over all temporal tree-child networks. To capture this number, we consider the weighted fork-picking sequences for a pair of phylogenetic trees, showing in Theorem 3 that, in case it exists, the rigid hybrid number of two phylogenetic trees is equal to the minimum taken over the weights of all possible fork-picking sequences for the two trees. This result can be regarded as analogue of Humphries et al. (2013a, Theorem 2).

In Sect. 8, we consider the relationship between the rigid hybrid number and three closely related hybrid numbers: the weak, beaded, and temporal hybrid numbers (the beaded hybrid number was implicitly defined in van Iersel et al. (2018)). In particular, in Theorem 4, we first show that there is a pair of phylogenetic trees on a set *X* with |*X*| arbitrarily large, so that the difference between the temporal and rigid hybrid numbers for these two trees is at least $\frac{|X|}{4}-3$. Then in Theorem 5, we show that there exist two phylogenetic trees whose beaded, weak, rigid, and temporal hybrid numbers are all distinct from one another. We conclude in Sect. 9 by presenting some open problems and discussing some possible future directions of research.

## Preliminaries

Let *G* denote a directed, acyclic graph with a single root, i.e., a vertex with indegree zero. Let *V*(*G*) denote the vertex set of *G*, *E*(*G*) the set of (directed) edges of *G*, and $\rho _G$ the unique root of *G*. A vertex in *G* with indegree one and outdegree zero is called a *leaf*; an edge of *G* incident with a leaf of *G* a *pendant edge* of *G*. Furthermore, we denote the set of all leaves of *G* by *L*(*G*).

Suppose $v\in V(G)$. We say that a vertex $u\in V(G)$ is *above* *v* if there exists a directed path from *u* to *v* (note that *u* could equal *v*). If *u* is above *v*, then we also write $u\preceq _G v$ or simply $u\preceq v$ if *G* is clear from the context. Furthermore, we say that *v* is *below* *u*. We call any vertex above *v* an *ancestor* of *v* and any vertex below *v* a *descendant* of *v*. Finally, we say that two distinct edges $e=(u,v)$ and $e'=(u',v')$ of *G* are *comparable* if *v* is above $u'$ or $v'$ is above *u*. Otherwise we say that *e* and $e'$ are *incomparable*.

Let *X* be a finite set of size at least 2. Following e.g. Huber et al. (2016, p.1764) a rooted, directed acyclic graph ${{\mathscr {N}}}$ is called a *phylogenetic network (on X)* if the following properties are satisfied:

1. the root $\rho _{{{\mathscr {N}}}}$ of ${{\mathscr {N}}}$ has indegree zero and outdegree two,
2. *X* is the set of leaves of ${{\mathscr {N}}}$, and
3. each remaining vertex of ${{\mathscr {N}}}$ has either indegree one and outdegree two, or indegree two and outdegree one.

Unless stated otherwise, phylogenetic networks do not contain parallel edges.

Now let ${{\mathscr {N}}}$ be a phylogenetic network. We refer to a vertex of ${{\mathscr {N}}}$ with indegree two and outdegree one as a *reticulation vertex*, and to a vertex with indegree one and outdegree zero or two as a *tree vertex*. The set of reticulation vertices of ${{\mathscr {N}}}$ is denoted by $Ret({{\mathscr {N}}})$. We put $h({{\mathscr {N}}}) = |Ret({{\mathscr {N}}})|$. Moreover, we call a directed path *P* from a vertex *v* to a leaf *x* in a phylogenetic network a *tree-path* if each vertex on *P*, except possibly *v*, is a tree vertex.

A *phylogenetic tree (on X)* is a phylogenetic network on *X* that does not have any reticulation vertices. We say that two phylogenetic trees ${{\mathscr {T}}}$ and ${{\mathscr {T}}}'$ on *X* are *isomorphic*, denoted by ${{\mathscr {T}}}\cong {{\mathscr {T}}}'$, if there exists a bijection $V({{\mathscr {T}}})\rightarrow V({{\mathscr {T}}}')$ that induces a graph isomorphism between ${{\mathscr {T}}}$ and ${{\mathscr {T}}}'$ that is the identity on *X*. If ${{\mathscr {T}}}$ is a phylogenetic tree on *X*, and $Y\subseteq X$, then the *last common ancestor of Y*, denote by $lca_{{{\mathscr {T}}}}(Y)$, is the unique vertex *v* of ${{\mathscr {T}}}$ that is an ancestor of every element in *Y* and there is no vertex $w\not =v$ such that *w* is a descendant of *v* and *w* is an ancestor of every element in *Y*. For any $2\le l\le |X|$ elements $x_1,\ldots , x_l\in X$, we sometimes also write $lca_{{{\mathscr {T}}}}(x_1,\ldots , x_l)$ rather than $lca_{{{\mathscr {T}}}}(\{x_1,\ldots , x_l\})$. Now let $Y'\subseteq V({{\mathscr {T}}})$. We denote by ${{\mathscr {T}}}(Y')$ the minimal subtree of ${{\mathscr {T}}}$ that connects all vertices in $Y'$ and by ${{\mathscr {T}}}|_{Y'}$ the tree obtained from ${{\mathscr {T}}}(Y')$ by suppressing all vertices that have indegree one and outdegree one. We refer to ${{\mathscr {T}}}|_{Y'}$ as the *restriction* of ${{\mathscr {T}}}$ to $Y'$. Note that, if $Y'$ is a subset of *X* such that $|Y'|\ge 2$, then the root of ${{\mathscr {T}}}(Y')$ equals $lca_{{{\mathscr {T}}}}(Y')$.

Suppose ${{\mathscr {N}}}$ is a phylogenetic network on *X*. Following Steel (2016), we say that ${{\mathscr {N}}}$ is *temporal* (Moret et al. 2004) if there exists a map $t : V({{\mathscr {N}}})\rightarrow {\mathbb {R}}^{\ge 0}$ such that, for all $(p,q)\in E({{\mathscr {N}}})$, we have $t(p) = t(q)$ whenever *q* is a reticulation vertex and $t(p) < t(q)$, otherwise. In that case, we call *t* a *temporal labelling* of ${{\mathscr {N}}}$. Unless of relevance to the discussion, we always omit the temporal labelling when depicting a temporal network. We say that ${{\mathscr {N}}}$ is *tree-child* (Cardona et al. 2009b) if, for each non-leaf vertex $v \in V({{\mathscr {N}}})$ at least one of the children of *v* is a tree vertex. Note that a tree-child network was called a phylogenetic network in Humphries et al. (2013a, p.1883) and that a temporal tree-child network (in our sense) was called a (binary) *time-consistent tree-child network*, or *TCTC-network* in Cardona et al. (2009a).

## Weakly displaying two trees in a network

To define and understand the notion of rigidly displaying, it is useful to first consider the more general notion of weakly displaying. As well as recalling the definition of weakly displaying, we derive some of its basic properties which will be useful later, and explain how the concept of rigidly displaying is related to the stronger notion of displaying (see Theorem 1).

Let ${{\mathscr {T}}}$ be a phylogenetic tree on *X* and let ${{\mathscr {N}}}$ be a phylogenetic network on *X*. Furthermore, let $\psi$ be a map that maps each vertex of ${{\mathscr {T}}}$ to a vertex of ${{\mathscr {N}}}$ and each edge $e=(u,v)$ of ${{\mathscr {T}}}$ to a directed path from the image of *u* under $\psi$ to the image of *v* under $\psi$. To distinguish the mapping of a vertex *v* from that of an edge *e* in ${{\mathscr {T}}}$, we use $\psi (v)$ to denote the vertex in ${{\mathscr {N}}}$ that *v* is mapped to under $\psi$ and $\psi [e]$ to denote the directed path in ${{\mathscr {N}}}$ that *e* is mapped to under $\psi$. We call $\psi$ a *display map* for ${{\mathscr {T}}}$ in ${{\mathscr {N}}}$ if the following properties hold:

(i) for each $$x\in X$$, $$\psi (x)=x$$,
(ii) for all $v\in V({{\mathscr {T}}})$, $\psi (v)$ is a tree vertex or the root of ${{\mathscr {N}}}$,
(iii) for each edge *e* of ${{\mathscr {T}}}$, $\psi [e]$ contains at least one edge of ${{\mathscr {N}}}$, and
(iv) for any two distinct edges $e=(u,v)$ and $e'=(u,v')$ of ${{\mathscr {T}}}$, the first edge of $\psi [e]$ is different from the first edge of $\psi [e']$ in ${{\mathscr {N}}}$.

Note that the definition of a display map $\psi$ is equivalent to $\psi$ being a locally separated reconciliation as defined in Huber et al. (2016, Section 7).

Now, let $\psi$ be a display map for ${{\mathscr {T}}}$ in ${{\mathscr {N}}}$, and let *P* be a directed path of ${{\mathscr {T}}}$. It follows immediately from the definition of $\psi$, that the edge set

$$\begin{aligned} \bigcup _{e\in E(P)}E(\psi [e]) \end{aligned}$$

induces a directed path in ${{\mathscr {N}}}$. We will freely use this fact throughout the remainder of the paper.

**Fig. 2:**
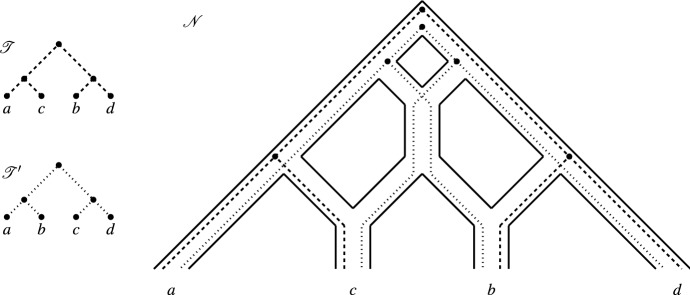
Two phylogenetic trees ${{\mathscr {T}}}$ and ${{\mathscr {T}}}'$ on $X=\{a,b,c,d\}$ that are weakly displayed by the network ${{\mathscr {N}}}$ on *X* via the indicated display maps $\psi$ and $\psi '$ for ${{\mathscr {T}}}$ and ${{\mathscr {T}}}'$, respectively, for which $\gamma _{\psi .\psi '}(w) \le 2$ holds for all $w \in V({{\mathscr {N}}})$. However, ${{\mathscr {T}}}$ and ${{\mathscr {T}}}'$ are *not* both displayed by ${{\mathscr {N}}}$ (the tree ${{\mathscr {T}}}'$ is not displayed)

Following Huber et al. (2016), we say that ${{\mathscr {T}}}$ is *weakly displayed* by ${{\mathscr {N}}}$ if there exists a (not necessarily unique) display map for ${{\mathscr {T}}}$ in ${{\mathscr {N}}}$. To reduce notation, we will sometimes not explicitly refer to the display map. Note that if ${{\mathscr {N}}}$ displays ${{\mathscr {T}}}$ as defined in the introduction then ${{\mathscr {N}}}$ also weakly displays ${{\mathscr {T}}}$. However, the converse is not necessarily true. For example, referring to Fig. 2, ${{\mathscr {T}}}'$ is weakly displayed by ${{\mathscr {N}}}$ for the indicated display map but ${{\mathscr {T}}}'$ is not displayed by ${{\mathscr {N}}}$.

The notion of weakly displayed was introduced in Huber et al. (2016) in terms of a construction that allows the unfolding of a phylogenetic network on *X* into a so-called multi-labelled tree on *X* (Huber and Moulton 2006). Such trees are similar to phylogenetic trees in that they have no vertices with in- and outdegree one and the root has indegree zero. However the requirement that the leaf-set is *X* is relaxed to the requirement that an element of *X* can label more than one leaf (which is not allowed in the case of phylogenetic trees).

Now, suppose that $\psi$ is a display map for ${{\mathscr {T}}}$ in ${{\mathscr {N}}}$. For any edge $e=(u,v)\in E({{\mathscr {T}}})$, we denote by $\psi [e]-\psi (u)$ the set of all vertices in $V({{\mathscr {N}}})$ that lie on $\psi [e]$ except for $\psi (u)$. Let $w \in V({{\mathscr {N}}})$, and let $e=(u,v)$ be an edge of ${{\mathscr {T}}}$. If $\psi (v)=w$, we say that $\psi [e]$ *ends at* *w* and if $w \in \psi [e]-\psi (u)$, but $\psi (v)\ne w$ we say that $\psi [e]$ *passes through* *w*. In addition, we define

$$\begin{aligned} \gamma _{\psi }(w)=|\{e=(u,v) \in E({{\mathscr {T}}}) \,:\, w \in \psi [e]-\psi (u) \}|, \end{aligned}$$

i.e., $\gamma _{\psi }(w)$ counts the number of edges in ${{\mathscr {T}}}$ such that their image under $\psi$ either ends or passes through *w* so that in particular $\gamma _{\psi }(\rho _{{{\mathscr {N}}}})=0$. Finally, if ${{\mathscr {T}}}'$ is an additional phylogenetic tree on *X* that is weakly displayed by ${{\mathscr {N}}}$ via a display map $\psi '$, then we put

$$\begin{aligned} \gamma (w)= \gamma _{\psi ,\psi '}(w)= \gamma _{\psi }(w)+\gamma _{\psi '}(w) \end{aligned}$$

(see e.g. Fig. 2). To reduce notation we sometimes drop the subscript in $\gamma _{\psi ,\psi '}$ as indicated if $\psi$ and $\psi '$ are clear from the context.

We now prove two lemmas about display maps which will be useful later.

### Lemma 1

Let ${{\mathscr {N}}}$ be a temporal tree-child network on *X* and let ${{\mathscr {T}}}$ be a phylogenetic tree on *X*. Then the following holds.

(i) ${{\mathscr {N}}}$ displays ${{\mathscr {T}}}$ if and only if there exists a display map $\psi$ for ${{\mathscr {T}}}$ in ${{\mathscr {N}}}$ such that, for all $w \in V({{\mathscr {N}}})$, we have $\gamma _{\psi }(w)\le 1$,
(ii) if $\psi$ is a display map for ${{\mathscr {T}}}$ in ${{\mathscr {N}}}$ as specified in Assertion (i) then $\psi (\rho _{{{\mathscr {T}}}}) = \rho _{{{\mathscr {N}}}}$.

### Proof

(i) Assume first that ${{\mathscr {N}}}$ displays ${{\mathscr {T}}}$. Then since a phylogenetic network on *X* that displays a phylogenetic tree on *X* clearly also weakly displays that tree, it is straight-forward to see that there exists a display map $\psi$ for ${{\mathscr {T}}}$ in ${{\mathscr {N}}}$ such that, for all $w \in V({{\mathscr {N}}})$, we have $\gamma _{\psi }(w)\le 1$.

Conversely, assume that there exists a display map $\psi$ for ${{\mathscr {T}}}$ in ${{\mathscr {N}}}$ such that, for all $w \in V({{\mathscr {N}}})$, we have $\gamma _{\psi }(w)\le 1$. Then, for each $v\in Ret({{\mathscr {N}}})$, there exists at most one edge $e\in E({{\mathscr {T}}})$ such that $\psi [e]$ passes through *v*. Note that since *v* is not a tree-vertex of ${{\mathscr {N}}}$, there cannot exist an edge in $e\in E({{\mathscr {T}}})$ such that $\psi [e]$ ends in *v*. Since ${{\mathscr {N}}}$ is tree-child it follows that the subgraph ${{\mathscr {N}}}'$ of ${{\mathscr {N}}}$ with vertex set $V({{\mathscr {N}}})$ and edge set $\bigcup _{e\in E({{\mathscr {T}}})} \psi [e]$ is a rooted tree with leaf set *X*. Furthermore, the outdegree of the root of ${{\mathscr {N}}}'$ must be two as ${{\mathscr {N}}}$ is temporal and, therefore, ${{\mathscr {N}}}$ cannot contain a *shortcut*, that is, if there exists a directed path from a vertex *u* to a vertex *v* in ${{\mathscr {N}}}$ which contains at least three vertices then there cannot also be the edge (*u*, *v*) in ${{\mathscr {N}}}$. Since ${{\mathscr {N}}}'$ is isomorphic to a subdivision of ${{\mathscr {T}}}$ it follows that ${{\mathscr {N}}}$ displays ${{\mathscr {T}}}$.

(ii) Assume for contradiction that $\psi (\rho _{{{\mathscr {T}}}})$ is not the root $\rho _{{{\mathscr {N}}}}$ of ${{\mathscr {N}}}$. Since ${{\mathscr {N}}}$ is temporal tree-child, the two children *u* and *v* of $\rho _{{{\mathscr {N}}}}$ must be two distinct tree vertices. Moreover, there must exist a tree-path $p_x$ in ${{\mathscr {N}}}$ from *u* to some leaf *x* and a tree-path $p_y$ from *v* to some leaf *y*. Note that since $p_x$ and $p_y$ cannot intersect, we must have $x\not = y$. Since $\psi$ is a display map for ${{\mathscr {T}}}$ in ${{\mathscr {N}}}$ that satisfies the properties of Assertion (i) and *x* and *y* are also leaves of ${{\mathscr {T}}}$ it follows that $lca_{{{\mathscr {T}}}}(x,y)$ is mapped to an ancestor of *x* and *y* in ${{\mathscr {N}}}$ under $\psi$ as ${{\mathscr {N}}}$ must display ${{\mathscr {T}}}$. Extending the paths $p_x$ and $p_y$ to tree-paths starting at $\rho _{{{\mathscr {N}}}}$ implies that this ancestor must be $\rho _{{{\mathscr {N}}}}$. Thus, $\psi (\rho _{{{\mathscr {T}}}}) = \rho _{{{\mathscr {N}}}}$. $\square$

To state the second lemma we require some further definitions. We call a subgraph ${{\mathscr {N}}}'$ of ${{\mathscr {N}}}$ a *pendant subnetwork* of ${{\mathscr {N}}}$ if there exists a tree vertex *v* in ${{\mathscr {N}}}$ such that when deleting the incoming edge of *v* the network decomposes into two connected components where the component that contains *v* in its vertex set is a phylogenetic network ${{\mathscr {N}}}'$ on $L({{\mathscr {N}}}')\subseteq L({{\mathscr {N}}})$. For technical reasons, we consider ${{\mathscr {N}}}$ to be a pendant subnetwork of itself. A *pendant subtree* of ${{\mathscr {N}}}$ is a pendant subnetwork of ${{\mathscr {N}}}$ that is a phylogenetic tree. Note that a pendant subnetwork and therefore also a pendant subtree must have at least two leaves. Also, note that in what follows, if ${{\mathscr {N}}}$ is a phylogenetic network that weakly displays a phylogenetic tree via a display map $\psi$ and ${{\mathscr {T}}}$ is a pendant subtree of ${{\mathscr {N}}}$, then in order to simplify notation we shall identify ${{\mathscr {T}}}$ with its pre-image under $\psi$.

### Lemma 2

Let ${{\mathscr {N}}}$ be a phylogenetic network on *X* that weakly displays two distinct phylogenetic trees ${{\mathscr {T}}}$ and ${{\mathscr {T}}}'$ on *X* via display maps $\psi$ and $\psi '$, respectively. Furthermore, let *v* be a tree vertex of ${{\mathscr {N}}}$. Then the following hold.

(i) If $\gamma _{\psi ,\psi '}(v) \ge 3$, then there is a vertex in $Ret({{\mathscr {N}}})$ which is an ancestor of *v* in ${{\mathscr {N}}}$.
(ii) If $\gamma _{\psi ,\psi '}(v)=2$ and *v* has a child that is the root of a pendant subtree ${{\mathscr {T}}}^*$ in ${{\mathscr {N}}}$, then the pre-image of ${{\mathscr {T}}}^*$ under $\psi$ and $\psi '$, respectively is also a pendant subtree of the respective tree ${{\mathscr {T}}}$ and ${{\mathscr {T}}}'$.

### Proof

(i) Suppose that *v* is a tree vertex of ${{\mathscr {N}}}$ such that $\gamma _{\psi ,\psi '}(v) \ge 3$. Then, without loss of generality, we may assume that ${{\mathscr {T}}}$ is such that $\gamma _{\psi }(v)\ge 2$. Hence, there are two distinct edges $e=(u,w)$ and $e'=(u',w')$ in ${{\mathscr {T}}}$ such that $v \in \psi [e]-\psi (u)$ and $v \in \psi [e']-\psi (u')$.

We show first that *e* and $e'$ are incomparable in ${{\mathscr {T}}}$. Indeed, assume for contradiction that *e* and $e'$ are comparable. Without loss of generality, we may assume that *w* is above $u'$ in ${{\mathscr {T}}}$. Then the edges on the directed path *P* in ${{\mathscr {T}}}$ starting at *u* and ending at $w'$ are collectively mapped by $\psi$ to a directed path $P_{e,e'}$ in ${{\mathscr {N}}}$ with edge set $\bigcup _{f\in E(P)}E(\psi [f])$. Note that $P_{e,e'}$ contains $\psi [e]$ and $\psi [e']$ as (directed) subpaths and that $\psi [e]$ cannot be a subpath of $\psi [e']$ and vice versa as otherwise Property (iii) in the definition of a display map cannot hold. Now, since $v \in \psi [e]-\psi (u)$ and $v \in \psi [e']-\psi (u')$ it follows that ${{\mathscr {N}}}$ contains a directed cycle; a contradiction. Hence, *e* and $e'$ are incomparable.

Let $q=lca_{{{\mathscr {T}}}}(u,u')$, and let *P* (resp. $P'$) be the directed path in ${{\mathscr {T}}}$ from *q* to *w* (resp. $w'$). Furthermore, let $P_e$ and $P_{e'}$ be the directed paths in ${{\mathscr {N}}}$ with edge sets

$$\begin{aligned} \bigcup _{f\in E(P)}E(\psi [f]) \text { and }\bigcup _{f\in E(P')}E(\psi [f]), \end{aligned}$$

respectively. As the first vertex on $P_e$ and $P_{e'}$ is $\psi (q)$, it follows from Property (iv) in the definition of a display map that the first edge on $P_e$ is different from the first edge on $P_{e'}$. Moreover, since *e* is an edge of *P* and $e'$ is an edge of $P'$, it follows that $\psi [e]$ is a subpath of $P_e$ and $\psi [e']$ is a subpath of $P_{e'}$. Hence, *v* is a vertex on $P_e$ and $P_{e'}$. Consequently, there is some vertex in $Ret({{\mathscr {N}}})$ which lies on $P_e$ and $P_{e'}$ and which is an ancestor of *v*.

(ii) Note that every leaf *x* in ${{\mathscr {T}}}^*$ must be contained in the image under $\psi$ of some directed path in ${{\mathscr {T}}}$ from the root of ${{\mathscr {T}}}$ to *x*, and similarly for ${{\mathscr {T}}}'$. Since, by assumption, $\gamma _{\psi ,\psi '}(v)=2$, there can be at most one such path in ${{\mathscr {T}}}$ and ${{\mathscr {T}}}'$, respectively, which has this property for every leaf in ${{\mathscr {T}}}^*$. Since the leaf set of ${{\mathscr {N}}}$, ${{\mathscr {T}}}$, and ${{\mathscr {T}}}'$, respectively, is *X* it follows that the pre-image of ${{\mathscr {T}}}^*$ under $\psi$ and $\psi '$, respectively, must be a pendant subtree of the respective tree ${{\mathscr {T}}}$ and ${{\mathscr {T}}}'$, as otherwise $\gamma _{\psi ,\psi '}(v)\ge 3$. $\square$

In Fig. 2, we presented an example where two phylogenetic trees ${{\mathscr {T}}}$ and ${{\mathscr {T}}}'$ are weakly displayed by the depicted phylogenetic network ${{\mathscr {N}}}$, $\gamma (w) \le 2$ for all $w \in V({{\mathscr {N}}})$ but ${{\mathscr {T}}}$ and ${{\mathscr {T}}}'$ are *not* both displayed by ${{\mathscr {N}}}$. So, in general, it does not suffice to insist that $\gamma (w) \le 2$ for all $w \in V({{\mathscr {N}}})$ for two phylogenetic trees to be displayed by a phylogenetic network. However, if we insist that the network is temporal tree-child, we now show that this condition actually suffices.

### Theorem 1

Suppose that ${{\mathscr {N}}}$ is a temporal tree-child network on *X* and that ${{\mathscr {T}}}$ and ${{\mathscr {T}}}'$ are two phylogenetic trees on *X* that are weakly displayed by ${{\mathscr {N}}}$ via display maps $\psi$ and $\psi '$, respectively. Then the following statements are equivalent.

(i) $${{\mathscr {N}}}$$ displays $${{\mathscr {T}}}$$ and $${{\mathscr {T}}}'$$.
(ii) $\gamma _{\psi ,\psi '}(v)=2$ for all $v \in V({{\mathscr {N}}})-\{\rho _{{{\mathscr {N}}}}\}$.
(iii) $\gamma _{\psi ,\psi '}(w)=2$ for all $w \in Ret({{\mathscr {N}}})$.

### Proof

(i) $\Rightarrow$ (ii) We show first that $\gamma _{\psi ,\psi '}(v)\ge 2$ must hold for all $v \in V({{\mathscr {N}}})-\{\rho _{{{\mathscr {N}}}}\}$. Assume for contradiction that there exists some vertex $v\in V({{\mathscr {N}}})$ such that $\gamma _{\psi ,\psi '}(v)\le 1$. Then one of $\gamma _{\psi }(v)=0$ or $\gamma _{\psi '}(v)=0$ must hold. Without loss of generality we may assume that $\gamma _{\psi }(v)=0$. Then there exists no edge $e\in E({{\mathscr {T}}})$ such that $\psi [e]$ either passes through *v* or ends in *v*. But then there cannot exist a leaf *x* of ${{\mathscr {N}}}$ that can be reached from *v* via a tree-path. Thus, ${{\mathscr {N}}}$ is not tree-child; a contradiction. Since, by assumption, ${{\mathscr {N}}}$ displays both ${{\mathscr {T}}}$ and ${{\mathscr {T}}}'$, Lemma 1 implies that $\gamma (v) \le 2$ for all $v \in V({{\mathscr {N}}})-\{\rho _{{{\mathscr {N}}}}\}$. Thus, $\gamma (v)=2$ must hold for all $v \in V({{\mathscr {N}}})-\{\rho _{{{\mathscr {N}}}}\}$.

(ii) $\Rightarrow$ (iii) This is trivial.

(iii) $\Rightarrow$ (i) By Lemma 1 it suffices to show that $\gamma _{\psi }(v)\le 1$ and $\gamma _{\psi '}(v)\le 1$ holds for all $v\in V({{\mathscr {N}}})$. Assume for contradiction that there exists some $v\in V({{\mathscr {N}}})$ and some tree in $\{{{\mathscr {T}}},{{\mathscr {T}}}'\}$, say ${{\mathscr {T}}}$, such that $\gamma _{\psi }(v)\ge 2$. Then $v\not =\rho _{{{\mathscr {N}}}}$. We claim that *v* is a tree vertex. Assume for contradiction that this is not the case. Then $v\in Ret({{\mathscr {N}}})$. Since ${{\mathscr {N}}}$ is tree-child there exists a leaf $x\in X$ below *v* that can be reached from *v* via a tree-path. Hence, any directed path from $\rho _{{{\mathscr {N}}}}$ to *x* must contain *v*. Thus, $\gamma _{\psi '}(v)\ge 1$ which, in turn, implies that $\gamma _{\psi ,\psi '}(v)=\gamma _{\psi }(v)+\gamma _{\psi '}(v)\ge 2+1=3$; a contradiction in view of Assertion (iii). Thus, *v* is a tree vertex, as claimed.

By Lemma 2(i), it follows that there must exist some vertex $w\in Ret({{\mathscr {N}}})$ that is an ancestor of *v*. Without loss of generality, we may assume that *w* is such that no vertex in $V({{\mathscr {N}}})$ distinct from *w* that is above *v* and below *w* is contained in $Ret({{\mathscr {N}}})$. Then there is a unique directed path *P* from *w* to *v* in ${{\mathscr {N}}}$. Since $\gamma _{\psi ,\psi '}(v) \ge 3$ and, $\gamma _{\psi ,\psi '}(w)=2$, there is a reticulation vertex contained in *P* that is not *w*; a contradiction to the choice of *w*. $\square$

### Remark 1

Note that using the same proofs it can be seen that both Lemma 1 and Theorem 1 also hold for the more general class of *normal* networks. These are phylogenetic networks that in addition to being tree-child do not contain a shortcut as defined in the proof of Lemma 1 (see e.g. McDiarmid et al. 2015, p. 208).

## Rigidly displaying two trees in a network

We now introduce the notion of rigidly displaying and present some of its basic properties. In Theorem 1, we showed that if ${{\mathscr {N}}}$ is a temporal tree-child network which weakly displays two phylogenetic trees ${{\mathscr {T}}}$ and ${{\mathscr {T}}}'$via display maps $\psi$ and $\psi '$, then in case $\gamma _{\psi ,\psi '}(v)=2$ for all $v \in V({{\mathscr {N}}})-\{\rho _{{{\mathscr {N}}}}\}$ it follows that ${{\mathscr {N}}}$ actually displays ${{\mathscr {N}}}$. To define rigidly displaying we relax this latter condition as follows.

We say that a phylogenetic network ${{\mathscr {N}}}$ on *X* *rigidly displays* two phylogenetic trees ${{\mathscr {T}}}$ and ${{\mathscr {T}}}'$ on *X* if ${{\mathscr {N}}}$ weakly displays ${{\mathscr {T}}}$ and ${{\mathscr {T}}}'$ via display maps $\psi$ and $\psi '$ respectively, for all $v \in Ret({{\mathscr {N}}})$ we have $\gamma _{\psi ,\psi '}(v) \le 3$ and, for each parent $w\in V({{\mathscr {N}}})$ of a reticulation vertex $v\in Ret({{\mathscr {N}}})$, we have $\gamma _{\psi ,\psi '}(w) \le 2$. For example, the network pictured in Fig. 1 rigidly displays the two phylogenetic trees depicted in that figure (where $\psi$ and $\psi '$ are the obvious display maps).

Note that, in contrast to the definitions of displaying and weakly displaying which refer to a single phylogenetic tree, rigidly displaying always refers to two phylogenetic trees. In addition, by Theorem 1 it follows that if ${{\mathscr {T}}}$ and ${{\mathscr {T}}}'$ are two phylogenetic trees on *X* that are displayed by a temporal tree-child network ${{\mathscr {N}}}$ on *X*, then ${{\mathscr {N}}}$ also rigidly displays ${{\mathscr {T}}}$ and ${{\mathscr {T}}}'$.

We conclude this section by presenting two technical lemmas concerning rigidly displaying trees in tree-child networks which we will use later.

### Lemma 3

Suppose ${{\mathscr {N}}}$ is a tree-child network on *X* and that ${{\mathscr {N}}}$ rigidly displays two phylogenetic trees ${{\mathscr {T}}}$ and ${{\mathscr {T}}}'$ on *X* via display maps $\psi$ and $\psi '$, respectively. Then $2 \le \gamma _{\psi ,\psi '}(v) \le 3$ for all $v \in V({{\mathscr {N}}})-\{\rho _{{{\mathscr {N}}}}\}$.

### Proof

Put $\gamma =\gamma _{\psi ,\psi '}$ and suppose $v \in V({{\mathscr {N}}})-\{\rho _{{{\mathscr {N}}}}\}$. Then since ${{\mathscr {N}}}$ rigidly displays ${{\mathscr {T}}}$ and ${{\mathscr {T}}}'$ it also weakly displays ${{\mathscr {T}}}$ and ${{\mathscr {T}}}'$. Since $v\not =\rho _{{{\mathscr {N}}}}$ and there exists some leaf below *v* that can be reached from *v* via a tree-path as ${{\mathscr {N}}}$ is tree-child, it follows in view of Property (ii) in the definition of a display map that $\gamma _{\psi }(v)\ge 1$ and that $\gamma _{\psi '}(v)\ge 1$. Hence, $2 \le \gamma (v)$.

For the remainder, assume for contradiction that there exists some $v\in V({{\mathscr {N}}})-\{\rho _{{{\mathscr {N}}}}\}$ such that $\gamma (v) \ge 4$. Then *v* must be a tree vertex of ${{\mathscr {N}}}$ as ${{\mathscr {N}}}$ rigidly displays ${{\mathscr {T}}}$ and ${{\mathscr {T}}}'$ and $\gamma (\rho _{{{\mathscr {N}}}})=0$. Let $P: v_1,v_2,\ldots ,v_k,v$ be a longest directed path of tree vertices in ${{\mathscr {N}}}$ that ends at *v*. Note that $\gamma (v_i)\ge \gamma (v)$, for all $1\le i\le k$. Also note that since $\rho _{{{\mathscr {N}}}}$ is not a tree vertex of ${{\mathscr {N}}}$, we cannot have $v_1= \rho _{{{\mathscr {N}}}}$. Let $w\in V({{\mathscr {N}}})$ denote the parent of $v_1$. Then $\gamma (w) \ge 4$. Hence, we cannot have $w=\rho _{{{\mathscr {N}}}}$. Since ${{\mathscr {N}}}$ rigidly displays ${{\mathscr {T}}}$ and ${{\mathscr {T}}}'$ it follows that *w* must be a tree vertex of ${{\mathscr {N}}}$. But then the extension of *P* by *w* results in a directed path of tree vertices of ${{\mathscr {N}}}$ that ends in *v* and that is longer than *P*; a contradiction. $\square$

**Fig. 3:**
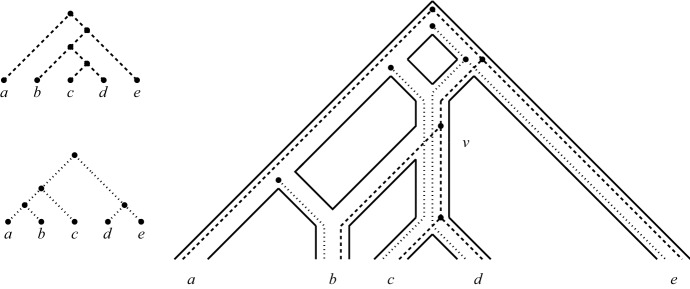
The two phylogenetic trees on the left are weakly displayed via the indicated display maps by the network depicted on the right. However, they are not rigidly displayed by the network for those maps because $\gamma (v)=3$ and *v* is the parent of a reticulation vertex

Note that the converse of the last lemma does not hold in general (see e. g. Fig. 3).

### Lemma 4

Suppose that ${{\mathscr {N}}}$ is a tree-child network on *X* that rigidly displays two phylogenetic trees ${{\mathscr {T}}}$ and ${{\mathscr {T}}}'$ on *X* and that $\psi$ is the underlying display map for ${{\mathscr {T}}}$ in ${{\mathscr {N}}}$. If $e=(u,v)$ is an edge of ${{\mathscr {T}}}$ such that $\psi [e]$ passes through a vertex $w\in Ret({{\mathscr {N}}})$, then $\psi (u)$ must be a parent of *w* in ${{\mathscr {N}}}$.

### Proof

Suppose $e=(u,v)$ is an edge in ${{\mathscr {T}}}$ such that $\psi [e]$ passes through a vertex $w \in Ret({{\mathscr {N}}})$. Assume for contradiction that $\psi (u)$ is not a parent of *w*. Let *p* be the parent of *w* in ${{\mathscr {N}}}$ such that *p* lies on $\psi [e]$. Then $\psi (u) \ne p$. As ${{\mathscr {N}}}$ is tree-child, there must be a tree-path in ${{\mathscr {N}}}$ starting at *p* and ending at some leaf $x \in X$. So, as *x* is a leaf of ${{\mathscr {T}}}$ and $\psi (u) \ne p$, there must be some edge $e' \ne e$ in ${{\mathscr {T}}}$ such that $\psi [e']$ passes through or ends at *p*. Moreover, considering the leaf *x* again, there must be an edge in ${{\mathscr {T}}}'$ which maps to a path in ${{\mathscr {N}}}$ via the underlying display map $\psi '$ for ${{\mathscr {T}}}'$ in ${{\mathscr {N}}}$ that either ends at or passes through *p*. It follows that $\gamma _{\psi ,\psi '}(p) \ge 3$; a contradiction as ${{\mathscr {N}}}$ rigidly displays ${{\mathscr {T}}}$ and ${{\mathscr {T}}}'$ and *p* is the parent of a vertex in $Ret({{\mathscr {N}}})$. $\square$

Note that, as the example of the two phylogenetic trees and the network in Fig. 2 shows, the assumption that ${{\mathscr {N}}}$ is tree-child is necessary for Lemma 4 to hold.

## Fork operations

In the next section we shall characterize when two phylogenetic trees are rigidly displayed by a temporal tree-child network in terms of sequences of certain operations on these trees. The basis for these sequences are fork-operations which we shall now introduce.

First, we recall that two leaves *x* and *y* of a phylogenetic tree ${{\mathscr {T}}}$ with $x\not =y$ are called a *cherry* of ${{\mathscr {T}}}$, denoted by $\{x,y\}$, if *x* and *y* have the same parent. Now, by a *fork* we mean a 2-leaved rooted tree (i. e. a cherry), a 3-leaved rooted tree (a *3-fork*) or a 4-leaved fully-balanced rooted tree (a *4-fork*). The following basic fact concerning forks is straight-forward to show.

### Lemma 5

Suppose ${{\mathscr {T}}}$ is a phylogenetic tree with $n\ge 3$ leaves. If $n=3$ then ${{\mathscr {T}}}$ is a 3-fork and if $n\ge 4$ then ${{\mathscr {T}}}$ must contain a pendant subtree that is either a 3-fork or a 4-fork.

**Fig. 4:**
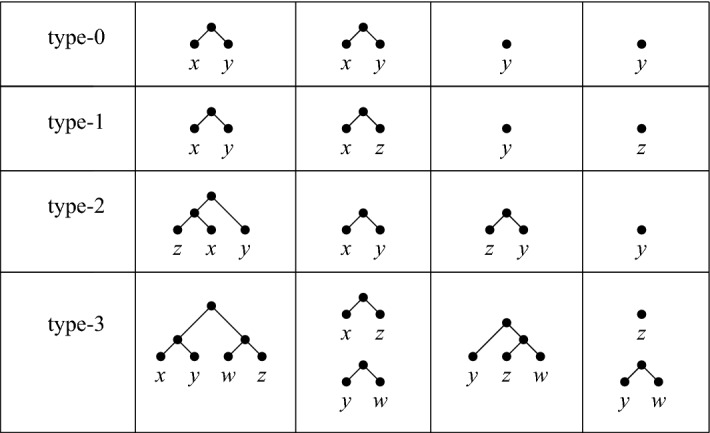
The four fork-operations each applied to leaf *x* in two phylogenetic trees, as defined in the text

**Fig. 5:**
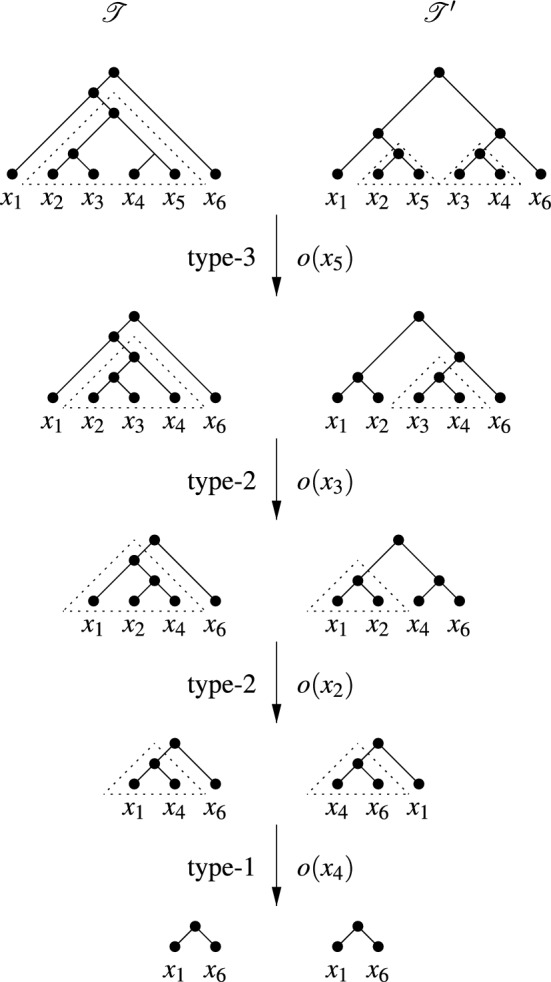
For the two pictured phylogenetic trees ${{\mathscr {T}}}$ and ${{\mathscr {T}}}'$ on $X=\{x_1,\ldots , x_6 \}$, we depict a fork-picking sequence $(S_1,C_2)$ for ${{\mathscr {T}}}$ and ${{\mathscr {T}}}'$ which has weight 1. In that sequence, $S_1$ is the special sequence $(o_3(x_5),$ $o_2(x_3), o_2(x_2), o_1(x_4))$, and $o_0(x_1)$ makes up $C_2$. The forks in ${{\mathscr {T}}}$ and ${{\mathscr {T}}}'$ to which a fork-operation is applied are indicated by dotted triangles

Now, a *(type*-*i*) *fork-operation* $o=o(x)$, $0 \le i \le 3$, is an operation that can be applied to a pair of phylogenetic trees ${{\mathscr {T}}}$ and ${{\mathscr {T}}}'$ on *X* for which there exists a leaf $x \in X$, together with a fork in each of ${{\mathscr {T}}}$ and ${{\mathscr {T}}}'$ containing *x* as depicted in the second and third columns of Fig. 4 (for example, for a type-2 operation one tree has a 3-fork ((*z*, *x*), *y*) and the other a cherry $\{x,y\}$ with $x,y,z \in X$ distinct). In the 4th and 5th columns of Fig. 4 the result of applying the operation *o*(*x*) to the two trees is pictured (for example, applying a type-2 operation to the 3-fork and the cherry in row 3 results in a phylogenetic tree with cherry $\{z,y\}$ and a phylogenetic tree whose cherry $\{x,y\}$ has been replaced by *y*). In particular, when we apply an operation *o* to some element $x\in X$, we remove the leaf *x* and its incident edge from both trees, and suppress any resulting vertices of degree 2, also removing the root and both edges incident with it in case $|X|=2$. In case the type *i* of the fork-operation is of importance we write $o_i(x)$ instead of *o*(*x*). We present an example of applying a sequence of fork-operations to two phylogenetic trees in Fig. 5.

Now, given two phylogenetic trees ${{\mathscr {T}}}$ and ${{\mathscr {T}}}'$ on the set $X=\{x_1,\ldots ,x_{n}\}$, $n\ge 3$, we call a sequence $(o(x_1),o(x_2),\ldots ,o(x_l))$ of *l* fork-operations, $1 \le l \le n-2$, a *special sequence* for ${{\mathscr {T}}}$ and ${{\mathscr {T}}}'$ if $o(x_l)$ is a type-1 operation on ${{\mathscr {T}}}|_{X- \{x_1,\ldots ,x_{l-1}\}}$ and ${{\mathscr {T}}}'|_{X - \{x_1,\ldots ,x_{l-1}\}}$ and, in case $l>1$, the following properties hold:

(i) There exists some ${{\mathscr {T}}}^* \in \{{{\mathscr {T}}},{{\mathscr {T}}}'\}$ such that each $o(x_i)$, $1 \le i \le l-1$, is a type-2 or a type-3 operation on ${{\mathscr {T}}}|_{X - \{x_1,\ldots ,x_{i-1}\}}$ and ${{\mathscr {T}}}'|_{X - \{x_1,\ldots ,x_{i-1}\}}$ and the associated 3- or 4-fork is a pendant subtree of ${{\mathscr {T}}}^*|_{X-\{x_1,\ldots ,x_{i-1}\}}$,
(ii) there exist two distinct elements $p,q\in X-\{x_1,x_2,\ldots ,x_l\}$ such that the last-but-one operation $o(x_{l-1})$ is a type-2 operation with fork $(p,(x_{l-1},x_l))$ and cherry $\{x_{l-1},p\}$ and the last operation is the type-1 operation $o(x_l)$ applied to the cherries $\{p,x_l\}$ and $\{q,x_l\}$, and
(iii) if $l>2$, then $x_i$ must be below $lca_{{{\mathscr {T}}}^*}(x_{l-1},x_l)$ for all $1 \le i \le l-2$ for the tree ${{\mathscr {T}}}^*$ in (i).

To illustrate this definition, consider the two phylogenetic trees ${{\mathscr {T}}}$ and ${{\mathscr {T}}}'$ on $X=\{x_1,x_2,\ldots ,x_6\}$ depicted in Fig. 5. Then $(o_3(x_5), o_2(x_3),o_2(x_2), o_1(x_4))$ is a special sequence for ${{\mathscr {T}}}$ and ${{\mathscr {T}}}'$ where, for example, $o_3(x_5)$ is a fork-operation of type-3 and the tree with cherry $(x_4,x_5)$ is the tree ${{\mathscr {T}}}^*$ mentioned in the definition of a fork-picking sequence. Note that an application of the last operation in a special sequence always results in phylogenetic trees with at least two leaves.

We conclude this section with an observation concerning special sequences which will be key to proving our main results.

### Proposition 1

Suppose that ${{\mathscr {N}}}$ is a temporal tree-child network on *X*, $|X|\ge 3$, that rigidly displays two phylogenetic trees ${{\mathscr {T}}}$ and ${{\mathscr {T}}}'$ on *X*. If no type-0 operation can be applied to ${{\mathscr {T}}}$ and ${{\mathscr {T}}}'$, then there is a special sequence $\sigma$ for ${{\mathscr {T}}}$ and ${{\mathscr {T}}}'$. Moreover, the two phylogenetic trees resulting from applying $\sigma$ to ${{\mathscr {T}}}$ and ${{\mathscr {T}}}'$ can be rigidly displayed by a temporal tree-child network ${{\mathscr {N}}}'$ with $h({{\mathscr {N}}}')=h({{\mathscr {N}}})-1 \ge 0$.

### Proof

Note first that $h({{\mathscr {N}}})>0$ as otherwise ${{\mathscr {N}}}$ would be a phylogenetic tree that is isomorphic with both ${{\mathscr {T}}}$ and ${{\mathscr {T}}}'$ implying that a type-0 operation can be applied to ${{\mathscr {T}}}$ and ${{\mathscr {T}}}'$; a contradiction.

Let $t:V({{\mathscr {N}}}) \rightarrow {\mathbb {R}}^{\ge 0}$ denote a temporal labelling for ${{\mathscr {N}}}$ and pick some $v \in Ret({{\mathscr {N}}})$ whose value is maximum under *t*. Let *u* and *w* be the parents of *v*. Since ${{\mathscr {N}}}$ does not contain parallel arcs, *u* and *w* are two distinct tree vertices of ${{\mathscr {N}}}$. Since ${{\mathscr {N}}}$ is tree-child, there must exist some child *p* of *u* that is not *v* and, similarly, there must exist a child *q* of *w* that is not *v*. We claim that *p* is a leaf of ${{\mathscr {N}}}$.

To see that this claim holds, assume for contradiction that *p* is the root of a pendant subgraph ${{\mathscr {N}}}^*$ of ${{\mathscr {N}}}$. Note that the choice of *v* implies that ${{\mathscr {N}}}^*$ is in fact a pendant subtree of ${{\mathscr {N}}}$. Moreover, Lemma 3 implies that there are display maps for ${{\mathscr {T}}}$ and ${{\mathscr {T}}}'$ in ${{\mathscr {N}}}$ such that $\gamma (u)=2$. By Lemma 2(ii) it follows that ${{\mathscr {N}}}^*$ is a pendant subtree of both ${{\mathscr {T}}}$ and ${{\mathscr {T}}}'$. Hence, ${{\mathscr {T}}}$ and ${{\mathscr {T}}}'$ have a common cherry and, so, we can apply a type-0 operation to ${{\mathscr {T}}}$ and ${{\mathscr {T}}}'$; a contradiction. Thus *p* must be a leaf of ${{\mathscr {N}}}$. Applying similar arguments to *q* implies that *q* must also be a leaf of ${{\mathscr {N}}}$.

Since ${{\mathscr {N}}}$ is tree-child, the choice of *v* implies that the child *s* of *v* is a leaf of ${{\mathscr {N}}}$ or the root of a pendant subtree of ${{\mathscr {N}}}$. Assume first that *s* is a leaf of ${{\mathscr {N}}}$. Then since ${{\mathscr {T}}}$ and ${{\mathscr {T}}}'$ are rigidly displayed by ${{\mathscr {N}}}$ and ${{\mathscr {T}}}$ and ${{\mathscr {T}}}'$ do not contain a common cherry, it is straight-forward to see using Lemma 4 that, without loss of generality, ${{\mathscr {T}}}$ and ${{\mathscr {T}}}'$ must contain the cherries $\{p,s\}$ and $\{q,s\}$, respectively. Hence we can apply a type-1 operation to *s*. This gives a special sequence of length 1 for ${{\mathscr {T}}}$ and ${{\mathscr {T}}}'$, from which the proposition follows.

So, suppose that *s* is the root of a pendant subtree ${{\mathscr {T}}}_1$ of ${{\mathscr {N}}}$, so that ${{\mathscr {T}}}_1$ has at least two leaves. Note first that $\gamma (v)=3$. Indeed, since ${{\mathscr {T}}}$ and ${{\mathscr {T}}}'$ are rigidly displayed by ${{\mathscr {N}}}$ and ${{\mathscr {N}}}$ is tree-child, we obtain $2\le \gamma (v)\le 3$ in view of Lemma 3. If $\gamma (v)=2$ held then ${{\mathscr {T}}}$ and ${{\mathscr {T}}}'$ would have a common cherry which implies that a type-0 operation can be applied to ${{\mathscr {T}}}$ and ${{\mathscr {T}}}'$; a contradiction. We can therefore assume without loss of generality that ${{\mathscr {T}}}_1$ is a pendant subtree of ${{\mathscr {T}}}$, and that this tree together with the leaf *p* also forms a pendant subtree of ${{\mathscr {T}}}$.

In case ${{\mathscr {T}}}_1$ has only two leaves *x* and *y*, say, then since $\gamma (v)=3$ it follows that ${{\mathscr {T}}}$ contains the 3-fork (*p*, (*x*, *y*)) and ${{\mathscr {T}}}'$ contains, without loss of generality, the cherries $\{p,y\}$ and $\{q,x\}$. Hence we can apply a type-2 operation to *y* and then apply a type-1 operation to *x* (since ${{\mathscr {T}}}|_{X-\{y\}}$ and ${{\mathscr {T}}}'|_{X-\{y\}}$ must contain the cherries $\{p,x\}$ and $\{q,x\}$, respectively). It follows that $(o_2(y),o_1(x))$ is a special sequence for ${{\mathscr {T}}}$ and ${{\mathscr {T}}}'$ since Property (i) of a special sequence holds for ${{\mathscr {T}}}^*={{\mathscr {T}}}$ and Property (ii) holds for *p* and *q* as defined above. The remainder of the proposition is a straight-forward consequence.

Assume for the remainder of the proof that ${{\mathscr {T}}}_1$ has at least two leaves. Put $Y=L({{\mathscr {T}}}_1)=\{x_1,\ldots , x_k\}$, $k\ge 2$. We claim that there exists a special sequence $\sigma =(o(x_1),\ldots , o(x_k))$ for ${{\mathscr {T}}}$ and ${{\mathscr {T}}}'$ with ${{\mathscr {T}}}^*={{\mathscr {T}}}$ and *p* and *q* as defined above. We prove the claim by induction on *k*.

Note that we have just shown that the claim holds for the base case $k=2$. So suppose the claim holds for all *k*, $k \ge 2$, and that $|Y|=k+1$. Note that as $k+1 \ge 3$, Lemma 5 implies that ${{\mathscr {T}}}_1$ contains either a 3-fork or a 4-fork.

Suppose ${{\mathscr {T}}}_1$ contains a 3-fork $\tau =(a,(b,c))$ where $a,b,c\in X$ are distinct. Then $\tau$ must be a pendant subtree of ${{\mathscr {T}}}$. As ${{\mathscr {T}}}$ and ${{\mathscr {T}}}'$ have no cherries in common and $\gamma (v)=3$, it follows that ${{\mathscr {T}}}'$ contains the cherry $\{a,b\}$ or the cherry $\{a,c\}$. Without loss of generality, we may assume that ${{\mathscr {T}}}'$ contains the cherry $\{a,c\}$. Hence, we can apply the type-2 operation *o*(*c*) to *c*. Note that this creates a cherry $\{a,b\}$ in ${{\mathscr {T}}}|_{X-\{c\}}$ which, in view of Property (iv) in the definition of a display map, is not a cherry in ${{\mathscr {T}}}'|_{X-\{c\}}$. Clearly, the network obtained from ${{\mathscr {N}}}$ by removing *c* and its incoming edge (suppressing the resulting indegree and outdegree one vertex) is temporal tree-child and rigidly displays ${{\mathscr {T}}}|_{X-\{c\}}$ and ${{\mathscr {T}}}'|_{X-\{c\}}$. By induction, we obtain a special sequence $\sigma _c=(o(x_1), o(x_2),\ldots , o(x_{k}))$ for ${{\mathscr {T}}}|_{X-\{c\}}$ and ${{\mathscr {T}}}'|_{X-\{c\}}$ with ${{\mathscr {T}}}^*={{\mathscr {T}}}|_{X-\{c\}}$ and *p* and *q* as defined above. We postulate that $(o_2(c),\sigma _c)$ is a special sequence for ${{\mathscr {T}}}$ and ${{\mathscr {T}}}'$ with ${{\mathscr {T}}}^*={{\mathscr {T}}}$ and *p* and *q* as defined above. Since *o*(*c*) is a type-2 operation applied to *c* it suffices to show that Property (iii) of a special sequence is satisfied for *c*, that is, *c* is below $lca_{{{\mathscr {T}}}}(x_k,x_{k-1})$. But this clearly holds since $b\in L({{\mathscr {T}}}_1|_{Y-\{c\}})$ and any ancestor of *b* is also an ancestor of *c* as $\{b,c\}$ is a cherry in $\tau$. Thus, $(o_2(c),\sigma _c)$ is a special sequence with the stated properties, as required.

Suppose ${{\mathscr {T}}}_1$ contains a 4-fork $\tau =((a,b),(c,d))$ where $a,b,c,d\in X$ are distinct. Then $\tau$ must again be a pendant subtree of ${{\mathscr {T}}}$. As ${{\mathscr {T}}}$ and ${{\mathscr {T}}}'$ have no cherries in common and $\gamma (v)=3$, it follows that, as before, we may assume without loss of generality that ${{\mathscr {T}}}'$ contains the cherries $\{a,c\}$ and $\{b,d\}$. Hence, we can apply the type-3 operation *o*(*c*) to *c*. Note that this creates a 3-fork (*d*, (*a*, *b*)) in ${{\mathscr {T}}}|_{X-\{c\}}$ and that $\{a,b\}$ is not a cherry in ${{\mathscr {T}}}'|_{X-\{c\}}$. Clearly, the network obtained from ${{\mathscr {N}}}$ by removing *c* and its incoming edge (suppressing the resulting indegree and outdegree one vertex) is again temporal tree-child and rigidly displays ${{\mathscr {T}}}|_{X-\{c\}}$ and ${{\mathscr {T}}}'|_{X-\{c\}}$. By induction, we obtain a special sequence $\sigma _c =(o(x_1), o(x_2),\ldots , o(x_k))$ for ${{\mathscr {T}}}|_{X-\{c\}}$ and ${{\mathscr {T}}}'|_{X-\{c\}}$ with ${{\mathscr {T}}}^*={{\mathscr {T}}}|_{X-\{c\}}$ and *p* and *q* as defined above. Employing similar arguments as in the previous case implies that $(o_3(c),\sigma _c)$ is a special sequence for ${{\mathscr {T}}}$ and ${{\mathscr {T}}}'$ with ${{\mathscr {T}}}^*={{\mathscr {T}}}$ and *p* and *q* as defined above. This completes the proof of the claim.

To complete the proof, note that as ${{\mathscr {T}}}_1$ is a pendant subtree of ${{\mathscr {N}}}$, we can remove ${{\mathscr {T}}}_1$ and *v* (plus all its incident edges) from ${{\mathscr {N}}}$, and suppress the resulting vertices of degree two to obtain a network ${{\mathscr {N}}}'$ with $h({{\mathscr {N}}}')=h({{\mathscr {N}}})-1 \ge 0$. As ${{\mathscr {N}}}$ rigidly displays ${{\mathscr {T}}}$ and ${{\mathscr {T}}}'$, it follows that ${{\mathscr {N}}}'$ rigidly displays their restrictions ${{\mathscr {T}}}|_{X-L({{\mathscr {T}}}_1)}$ and ${{\mathscr {T}}}'|_{X-L({{\mathscr {T}}}_1)}$. Moreover, as ${{\mathscr {N}}}$ is tree-child and $v\in Ret({{\mathscr {N}}})$, we have that ${{\mathscr {N}}}'$ is also tree-child. Since *p* and *q* are leaves of ${{\mathscr {N}}}$ and ${{\mathscr {N}}}$ is temporal, it follows that ${{\mathscr {N}}}'$ is temporal. $\square$

## Fork-picking sequences

In this section we characterize when two phylogenetic trees are rigidly displayed by a temporal tree-child network, in terms of a generalization of special sequences which we now introduce. Suppose that ${{\mathscr {T}}}$ and ${{\mathscr {T}}}'$ are two phylogenetic trees on $X=\{x_1,\ldots , x_n\}$ where $n\ge 2$ and that $\sigma =(o(x_1),o(x_2),\ldots ,o(x_{n-1}))$ is a sequence of fork-operations for ${{\mathscr {T}}}$ and ${{\mathscr {T}}}'$ where $o(x_i)$ is on ${{\mathscr {T}}}|_{X- \{x_1,\ldots ,x_{i-1}\}}$ and ${{\mathscr {T}}}'|_{X- \{x_1,\ldots ,x_{i-1}\}}$, for all $1\le i\le n-1$. Then we call $\sigma$ a *fork-picking sequence for* ${{\mathscr {T}}}$ *and* ${{\mathscr {T}}}'$ if $\sigma$ is of the form $(C_1,S_1,C_2,S_2,\ldots , C_k,S_k,C_{k+1})$, some $k\ge 0$, such that

(i) for all $1\le i\le k+1$, we have that $C_i$ is a possibly empty (except in case $i=k+1$) sequence of type-0 operations, and
(ii) for all $1\le i\le k$, we have that $S_i$ is a special sequence for ${{\mathscr {T}}}|_Y$ and ${{\mathscr {T}}}'|_Y$, where $Y=\{x_p, x_{p+1}, \dots , x_n\}$ and $o(x_p)$ is the first operation in $S_i$ (so that, in particular, $n-p+1\ge 3$).

To ease readability, we omit all those $C_i$ that are empty when writing down fork-picking sequences. Note that it follows from the definition that any fork-picking sequence can be decomposed in a unique way into the form $(C_1,S_1,C_2,S_2,\ldots , C_k,S_k,C_{k+1})$, and that all of the subsequences $S_i$ are non-empty.

To illustrate this definition, note that $\sigma ^*=(o_3(x_5),o_2(x_3),o_2(x_2), o_1(x_4),o_0(x_1))$ is a fork-picking sequence for the two phylogenetic trees depicted in Fig. 5, since it is of the form $(C_1,S_1,C_2)$ where $S_1 = (o_3(x_5),o_2(x_3), o_2(x_2), o_1(x_4))$ is the special sequence for ${{\mathscr {T}}}$ and ${{\mathscr {T}}}'$ considered in the previous section, $C_2= (o_0(x_1))$, and $C_1$ is the empty sequence.

We now provide a link between $h({{\mathscr {N}}})$ for a temporal tree-child network ${{\mathscr {N}}}$ that rigidly displays two phylogenetic trees and fork-picking sequences for these trees. We define the *weight* $w(\sigma )$ of a fork-picking sequence $\sigma$ to be the number of special sequences in $\sigma$ (or, equivalently, the number of type-1 operations in $\sigma$ since any special sequence contains precisely one type-1 operation).

### Proposition 2

Suppose that ${{\mathscr {N}}}$ is a temporal tree-child network on *X* that rigidly displays two phylogenetic trees ${{\mathscr {T}}}$ and ${{\mathscr {T}}}'$ on *X*. Then there is a fork-picking sequence $\sigma$ for ${{\mathscr {T}}}$ and ${{\mathscr {T}}}'$ with $h({{\mathscr {N}}}) \ge w(\sigma )$.

### Proof

Clearly, the theorem holds for $|X|=2$. So assume that $|X|\ge 3$. We prove the theorem by induction on $h({{\mathscr {N}}})$. If $h({{\mathscr {N}}})=0$, then ${{\mathscr {N}}}$, ${{\mathscr {T}}}$, and ${{\mathscr {T}}}'$ are all isomorphic to one another. But then we can take a fork-picking sequence $\sigma$ for ${{\mathscr {T}}}$ and ${{\mathscr {T}}}'$ consisting solely of type-0 operations (i.e. $\sigma =(C_1)$), and so $w({{\mathscr {N}}})=0=h({{\mathscr {N}}})$.

Now, assume that $h({{\mathscr {N}}})=k$, some $k>0$, and that the theorem holds for all temporal tree-child networks ${{\mathscr {N}}}'$ with $0\le h({{\mathscr {N}}}')<k$.

Apply type-0 operations to ${{\mathscr {T}}}$ and ${{\mathscr {T}}}'$ until no more can be applied. If the resulting sequence $C_1$ of operations has length $|X|-1$, then it is a fork-picking sequence for ${{\mathscr {T}}}$ and ${{\mathscr {T}}}'$ and $w(\sigma )=0< k =h({{\mathscr {N}}})$. Hence, the theorem holds. Otherwise, let ${{\mathscr {T}}}_1$ and ${{\mathscr {T}}}_1'$ be the phylogenetic trees resulting after applying the operations in $C_1$, noting that $|L({{\mathscr {T}}}_1)|=|L({{\mathscr {T}}}'_1)| \ge 3$.

Since by construction no type-0 operation can be applied to ${{\mathscr {T}}}_1$ and ${{\mathscr {T}}}_1'$, by Proposition 1 it follows that there is a special sequence $S_1$ for ${{\mathscr {T}}}_1$ and ${{\mathscr {T}}}_1'$, and that the two phylogenetic trees ${{\mathscr {T}}}_2$ and ${{\mathscr {T}}}_2'$ resulting from applying $S_1$ can be rigidly displayed by a temporal tree-child network ${{\mathscr {N}}}'$ with $h({{\mathscr {N}}}')=h({{\mathscr {N}}})-1 \ge 0$.

It follows by induction that there is a fork-picking sequence $\sigma '= (C_1',S_1',\ldots ,C'_{k'+1})$, some $k'\ge 0$, for ${{\mathscr {T}}}_2$ and ${{\mathscr {T}}}_2'$ such that $k-1=h({{\mathscr {N}}}')\ge w(\sigma ')=k'$. Hence, $\sigma = (C_1,S_1,C'_1,S_1',\ldots ,C'_{{k'}+1})$ is a fork-picking sequence for ${{\mathscr {T}}}$ and ${{\mathscr {T}}}'$ such that $h({{\mathscr {N}}})=k\ge w(\sigma )$. $\square$

Now, as defined in Humphries et al. (2013a), we say for $n=|X|$ that an ordering $\sigma = (x_1 , x_2, \ldots , x_{n-1},x_n)$ of *X* is a *cherry-picking sequence* for two phylogenetic trees ${{\mathscr {T}}}$ and ${{\mathscr {T}}}'$ on *X* if, for all $1\le i\le n-1$, $x_i$ is contained in a cherry in both ${{\mathscr {T}}}_i={{\mathscr {T}}}|_{X-\{x_1,\ldots , x_{i-1}\}}$ and ${{\mathscr {T}}}_i'={{\mathscr {T}}}'|_{X-\{x_1,\ldots , x_{i-1}\}}$. In addition, the *cherry-count* $c_i(\sigma )\in \{0,1\}$ *associated to* $x_i$ is 1 if the cherries in ${{\mathscr {T}}}_i$ and ${{\mathscr {T}}}'_i$ containing $x_i$ are different and 0 else.

Note that every cherry-picking sequence $\sigma =(x_1,x_2,\ldots ,x_n)$ for two phylogenetic trees ${{\mathscr {T}}}$ and ${{\mathscr {T}}}'$ on *X* gives rise to a fork-picking sequence for ${{\mathscr {T}}}$ and ${{\mathscr {T}}}'$. Namely, we make a sequence of operations $(o(x_1),\ldots ,o(x_{n-1}))$ with a type-1 operation applied to $x_i$ if $c_i(\sigma )=1$ and a type-0 operation applied to $x_i$ if $c_i(\sigma )=0$. In addition, any fork-picking sequence $(o(x_1),\ldots ,o(x_{n-1}))$ for two phylogenetic trees ${{\mathscr {T}}}$ and ${{\mathscr {T}}}'$ on *X* clearly gives rise to the cherry-picking sequence $(x_1,x_2,\ldots ,x_n)$. For example, the cherry-picking sequence $(x_5,x_3,x_2,x_4,x_1,x_6)$ with cherry counts (1, 1, 1, 1, 0, 0) arises from the fork-picking sequence $\sigma ^*$ given at the beginning of this section for the two phylogenetic trees in Fig. 5. Using these observations we obtain the following result.

### Theorem 2

Suppose that ${{\mathscr {T}}}$ and ${{\mathscr {T}}}'$ are two phylogenetic trees on *X*. Then the following statements are equivalent.

(i) ${{\mathscr {T}}}$ and ${{\mathscr {T}}}'$ are rigidly displayed by a temporal tree-child network on *X*.
(ii) ${{\mathscr {T}}}$ and ${{\mathscr {T}}}'$ are displayed by a temporal tree-child network on *X*.
(iii) there is a cherry-picking sequence for ${{\mathscr {T}}}$ and ${{\mathscr {T}}}'$.
(iv) there is a fork-picking sequence for ${{\mathscr {T}}}$ and ${{\mathscr {T}}}'$.

### Proof

(ii) $\Rightarrow$ (i) If two phylogenetic trees are displayed by a phylogenetic network then they are rigidly displayed by that network.

(iii) $\Rightarrow$ (ii) Apply (Humphries et al. 2013a, Theorem 1), which states that two phylogenetic trees are displayed by a temporal tree-child network if and only if there is a cherry-picking sequence for them.

(i) $\Rightarrow$ (iv) Apply Proposition 2.

(iv) $\Rightarrow$ (iii) Apply the observation stated before the statement of the corollary i. e. , that a fork-picking sequence gives rise to a cherry-picking sequence. $\square$

Note that the temporal tree-child networks whose existence is guaranteed in Theorem 2(i) and (ii) need not be the same.

Theorem 2 also sheds light on the following decision problem:

Rigidly Displaying

**Input**: Two phylogenetic trees ${{\mathscr {T}}}$ and ${{\mathscr {T}}}'$ on *X*.

**Output**: Does there exist a temporal tree-child network on *X* that rigidly displays ${{\mathscr {T}}}$ and ${{\mathscr {T}}}'$?

Indeed, Theorem 2 and the main result in Döcker et al. (2019, Theorem 1) (which states that it is NP-complete to decide whether or not there is a cherry-picking sequence for two phylogenetic trees) immediately imply:

### Corollary 1

The decision problem Rigidly Displaying is NP-complete.

## The rigid hybrid number of two trees

Suppose that there is some fork-picking sequence for two phylogenetic trees ${{\mathscr {T}}}$ and ${{\mathscr {T}}}'$ on *X* (or equivalently by Theorem 2, ${{\mathscr {T}}}$ and ${{\mathscr {T}}}'$ are rigidly displayed by some temporal tree-child network on *X*). We define

$$\begin{aligned} s_r({{\mathscr {T}}},{{\mathscr {T}}}') = \min \{w(\sigma ) \,:\, \sigma \text{ is } \text{ a } \text{ fork-picking } \text{ sequence } \text{ for } {{\mathscr {T}}} \text{ and } {{\mathscr {T}}}'\}, \end{aligned}$$

and

$$\begin{aligned} h_r({{\mathscr {T}}},{{\mathscr {T}}}') = \min \{h({{\mathscr {N}}}) \,:\, {{\mathscr {N}}} \text{ is } \text{ temporal } \text{ tree-child } \text{ and } \text{ rigidly } \text{ displays } {{\mathscr {T}}} \text{ and } {{\mathscr {T}}}'\}. \end{aligned}$$

We call $h_r({{\mathscr {T}}},{{\mathscr {T}}}')$ the *rigid (temporal tree-child) hybrid number* for ${{\mathscr {T}}}$ and ${{\mathscr {T}}}'$.

In this section, we prove:

### Theorem 3

If two phylogenetic trees ${{\mathscr {T}}}$ and ${{\mathscr {T}}}'$ on *X* are rigidly displayed by some temporal tree-child network on *X*, then $s_r({{\mathscr {T}}},{{\mathscr {T}}}')=h_r({{\mathscr {T}}},{{\mathscr {T}}}')$.

This theorem is an immediate consequence of Proposition 2 and the following result.

### Proposition 3

Suppose ${{\mathscr {T}}}$ and ${{\mathscr {T}}}'$ are two phylogenetic trees on *X* and that $\sigma$ is a fork-picking sequence for ${{\mathscr {T}}}$ and ${{\mathscr {T}}}'$. Then there exists a temporal tree-child network ${{\mathscr {N}}}_{\sigma }$ on *X* which rigidly displays ${{\mathscr {T}}}$ and ${{\mathscr {T}}}'$ and such that $w(\sigma ) \ge h({{\mathscr {N}}}_{\sigma })$.

### Proof

We establish the theorem using induction on $w(\sigma )$.

If $w(\sigma )=0$ then $\sigma =(C_1)$, where $C_1$ consists solely of type-0 operations. Hence ${{\mathscr {T}}}$ and ${{\mathscr {T}}}'$ are isomorphic and the required temporal tree-child network ${{\mathscr {N}}}_{\sigma }$ is given by ${{\mathscr {T}}}$.

Now suppose that $\sigma$ is a fork-picking sequence for ${{\mathscr {T}}}$ and ${{\mathscr {T}}}'$ with weight $w(\sigma )=k$, some $k>0$, and that, for any two phylogenetic trees ${{\mathscr {T}}}_1$ and ${{\mathscr {T}}}_1'$, the theorem holds for all fork-picking sequences $\sigma '$ for ${{\mathscr {T}}}_1$ and ${{\mathscr {T}}}_1'$ with $w(\sigma ')< k$.

As $w(\sigma )=k$, $\sigma$ is of the form $(C_1,S_1,C_2,S_2,\ldots ,S_k,C_{k+1})$. Let *Y* be the set of elements *y* in *X* such that *o*(*y*) is *not* in the sequence $(C_1,S_1)$. Then, as $C_{k+1}$ is not the empty sequence, $\sigma '= (C_2,S_2,\ldots ,S_k,C_{k+1})$ is a fork-picking sequence for ${{\mathscr {T}}}|_Y$ and ${{\mathscr {T}}}'|_Y$, with $w(\sigma ')=k-1$. By induction, it follows that there is a temporal tree-child network ${{\mathscr {N}}}'$ with $h({{\mathscr {N}}}') \le w(\sigma ')$ that rigidly displays ${{\mathscr {T}}}|_Y$ and ${{\mathscr {T}}}'|_Y$. Let $t'$ denote a temporal labelling for ${{\mathscr {N}}}'$.

**Fig. 6:**
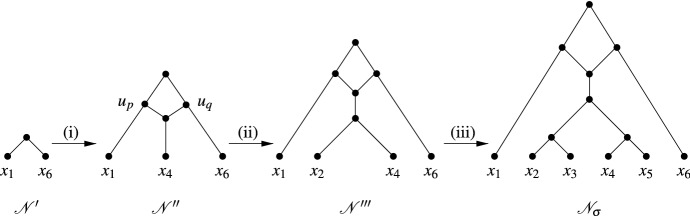
The construction of the temporal tree-child network ${{\mathscr {N}}}_{\sigma }$ for the fork-picking sequence $\sigma ^*=(C_1,S_1, C_2)$ for the phylogenetic trees ${{\mathscr {T}}}$ and ${{\mathscr {T}}}'$ in Fig. 5 as described in the proof of Proposition 3. Note that $C_1$ is the empty sequence. (i) Insertion of $x_4$. (ii) Insertion of $x_2$. (iii) Insertion of $x_3$ and $x_5$

We now construct a temporal tree-child network ${{\mathscr {N}}}_{\sigma }$ from ${{\mathscr {N}}}'$. We first consider the case that $C_1$ is the empty sequence. We illustrate this case in Fig. 6 in terms of the fork-picking sequence considered in Fig. 5.

Let $Y_1=\{y_1,\ldots , y_l\}$, $l\ge 1$, be such that $S_1=(o(y_1),\ldots ,o(y_l))$. To ease notation, put $x=y_l$ and, if $l\ge 2$, $y= y_{l-1}$.

Assume first that $l=1$. Since *o*(*x*) is a type-1 operation, there exist $p\not =q\in Y$ such that, without loss of generality, $\{p, x\}$ is a cherry in ${{\mathscr {T}}}|_{Y\cup \{x\}}$ and $\{q, x\}$ is a cherry in ${{\mathscr {T}}}'|_{Y\cup \{x\}}$. Subdivide the pendant edges in ${{\mathscr {N}}}'$ incident with *p* and *q* by adding new vertices $u_p$ and $u_q$, respectively. Also, add in the leaf *x* below a newly added reticulation vertex *v* which has parents $u_p$ and $u_q$. Denote the resulting phylogenetic network by ${{\mathscr {N}}}''$. Clearly, since ${{\mathscr {N}}}'$ is tree-child we also have that ${{\mathscr {N}}}''$ is tree-child. Returning to the fork-picking sequence example considered in Fig. 5, we have for Fig. 6(i) that $Y=\{x_1,x_6\}$ and that ${{\mathscr {T}}}|_Y$, ${{\mathscr {T}}}'|_Y$ and ${{\mathscr {N}}}'$ are all isomorphic. Also $y=x_2$, $x=x_4$, $p=x_1$, and $q=x_6$.

For $w_p$ and $w_q$ the parents of $u_p$ and $u_q$ in ${{\mathscr {N}}}'$, respectively, choose some value $t''(u_q)$ with $\max \{t'(w_p),t'(w_q)\}<t''(u_q) <\min \{t'(p), t'(q)\}$ and put $t''(u_q)=t''(u_p)=t''(v)$. Also, choose some $t''(x)$ such that $t''(v) <t''(x)$ so that, together with $t'$, we obtain a temporal labelling $t''$ for ${{\mathscr {N}}}''$. Clearly, ${{\mathscr {N}}}''$ rigidly displays ${{\mathscr {T}}}|_{Y\cup \{x\}}$ and ${{\mathscr {T}}}'|_{Y\cup \{x\}}$. Putting ${{\mathscr {N}}}_{\sigma }={{\mathscr {N}}}''$ this completes the proof of the theorem in case $l=1$ since $h({{\mathscr {N}}}'')=h({{\mathscr {N}}}')+1\le w(\sigma ')+1 =w(\sigma )$.

Assume now that $l=2$. We explain how to insert *y* into ${{\mathscr {N}}}''$. By definition of a special sequence, *o*(*y*) is a type-2 operation. Without loss of generality, we may assume that ${{\mathscr {T}}}|_{Y\cup \{x,y\}}$ has a fork (*p*, (*x*, *y*)) and that ${{\mathscr {T}}}'|_{Y\cup \{x,y\}}$ has a cherry $\{y,p\}$ with *p* as in operation $o(y_l)$. Then we can construct a new temporal tree-child network ${{\mathscr {N}}}'''$ which rigidly displays ${{\mathscr {T}}}|_{Y\cup \{x,y\}}$ and ${{\mathscr {T}}}'|_{Y\cup \{x,y\}}$ by inserting a pendant edge incident with *y* via a subdivision vertex *s* on the pendant edge of ${{\mathscr {N}}}''$ incident with *x* to form a cherry $\{x,y\}$ in ${{\mathscr {N}}}'''$. Define the temporal labelling $t'''$ of ${{\mathscr {N}}}'''$ by putting $t'''(y)=t''(x)$, choosing some value $t'''(s)$ with $t''(v)< t'''(s)< t''(x)$, and appropriately using $t''$. Note that $h({{\mathscr {N}}}''')=h({{\mathscr {N}}}'')$ and so the theorem is also proven for ${{\mathscr {N}}}_{\sigma }={{\mathscr {N}}}''$ if $l=2$.

Assume next that $l\ge 3$. Suppose that we have a special sequence. Also, suppose that we have created a temporal tree-child network ${{\mathscr {N}}}^*$ from ${{\mathscr {N}}}'''$ by successively inserting, for some $i\ge 0$ all leaves $y_j$, $i+1 \le j \le l-2$, below the parent of the cherry $\{x,y\}$ in ${{\mathscr {N}}}'''$ to create a pendant subtree ${\overline{{{\mathscr {T}}}}}$ of ${{\mathscr {N}}}^*$ with leaf set $Y_2=\{y_{i+1},\ldots ,y_{l-2}, y,x\} \subseteq Y_1$ so that ${{\mathscr {N}}}^*$ rigidly displays the phylogenetic trees ${{\mathscr {T}}}|_{Y\cup Y_2}$ and ${{\mathscr {T}}}'|_{Y\cup Y_2}$. Without loss of generality, we may assume that ${{\mathscr {T}}}$ is the tree ${{\mathscr {T}}}^*$ in the definition of a special sequence for ${{\mathscr {T}}}$ and ${{\mathscr {T}}}'$.

We now show how to insert $y_i$ into ${{\mathscr {N}}}^*$. Consider operation $o(y_i)$. Then, by definition of a special sequence, $o(y_i)$ is either a type-2 operation or a type-3 operation, for which the 3-fork and 4-fork, respectively, is a pendant subtree of ${{\mathscr {T}}}_1={{\mathscr {T}}}|_{Y\cup Y_2\cup \{y_i\}}$ and, in view of Property (iii) of a special sequence, $y_i$ is below $lca_{{{\mathscr {T}}}}(y,x)$. Put ${{\mathscr {T}}}_1'={{\mathscr {T}}}'|_{Y\cup Y_2\cup \{y_i\}}$.

If $o(y_i)$ is a type-2 operation, then let $(a,(y_i,b))$ denote the 3-fork of ${{\mathscr {T}}}_1$ where $a,b\in X-\{y_i\}$ distinct. Then $\{a,b\}$ must be a cherry in ${{\mathscr {T}}}|_{Y\cup Y_2}$. Since $y_i$ is below $lca_{{{\mathscr {T}}}}(x,y)$, it follows that $\{a,b\}$ is a cherry in ${\overline{{{\mathscr {T}}}}}$. We can therefore first insert $y_i$ into the pendant edge of ${{\mathscr {N}}}^*$ incident with *b* and then extend the temporal labelling of ${{\mathscr {N}}}^*$ so as to obtain a temporal tree-child network that rigidly displays ${{\mathscr {T}}}_1$ and ${{\mathscr {T}}}'_1$.

If $o(y_i)$ is a type-3 operation, then let $((a,y_i),(b,c))$ denote the 4-fork in ${{\mathscr {T}}}_1$ where $a,b,c\in X-\{y_i\}$ are pairwise distinct. Then (*a*, (*b*, *c*)) must be a 3-fork in ${{\mathscr {T}}}_1|_{Y\cup Y_2}$. As $y_i$ is below $lca_{{{\mathscr {T}}}}(x,y)$ it follows that this 3-fork must also be a pendant subtree of ${\overline{{{\mathscr {T}}}}}$. We can therefore first insert $y_i$ into the pendant edge of ${{\mathscr {N}}}^*$ incident with *a* and then extend the temporal labelling of ${{\mathscr {N}}}^*$ so as to obtain a temporal network that rigidly displays ${{\mathscr {T}}}_1$ and ${{\mathscr {T}}}'_1$. Again for the fork-picking sequence example considered in Fig. 5, we have for Fig. 6(iii) that $o(x_3)$ is a type-2 operation with $a=x_4$ and $b=x_2$ and $o(x_5)$ is a type-3 operation with $a=x_4$ and $\{b,c\}=\{x_2,x_3\}$.

In summary, we can insert all of the elements of *Y* into ${{\mathscr {N}}}'$ in this way until we obtain a temporal tree-child network ${{\mathscr {N}}}_{\sigma }$ with $h({{\mathscr {N}}}_{\sigma })= h({{\mathscr {N}}}')+1$ which rigidly displays ${{\mathscr {T}}}$ and ${{\mathscr {T}}}'$. It follows by induction that

$$\begin{aligned} w(\sigma ) = w(\sigma ')+1 \ge h({{\mathscr {N}}}') +1 = h({{\mathscr {N}}}_{\sigma }), \end{aligned}$$

which completes the proof of the theorem in case $C_1$ is empty.

Now, suppose $C_1$ is not empty. Then we first insert all elements of $Y_1$ into ${{\mathscr {N}}}'$ as described in the case that $C_1$ is the empty sequence above to obtain a network ${{\mathscr {N}}}_1$ which rigidly displays ${{\mathscr {T}}}|_{Y\cup Y_1}$ and ${{\mathscr {T}}}'|_{Y\cup Y_1}$ and for which $w(\sigma ) \ge h({{\mathscr {N}}}_1)$ holds. Into ${{\mathscr {N}}}_1$ we then insert all elements $z\in X$ for which $o_0(z)$ is contained in $C_1$ in the (reverse) order specified by $C_1$, and at each step creating a cherry specified by *o*(*z*), to obtain a new temporal tree-child network ${{\mathscr {N}}}_{\sigma }$ which rigidly displays ${{\mathscr {T}}}$ and ${{\mathscr {T}}}'$. Clearly, $h({{\mathscr {N}}}_{\sigma })= h({{\mathscr {N}}}_1)$. Since $\sigma ''=(C_1,S_1,C_2,S_2,\ldots ,S_k,C_{k+1})$ is a fork-picking sequence for ${{\mathscr {T}}}$ and ${{\mathscr {T}}}'$ and $w(\sigma )=w(\sigma '')$ the theorem holds in this case too. $\square$

## The relationship between the rigid, temporal, weak and beaded hybrid numbers

As mentioned in the introduction, there are various ways to define hybrid numbers for two phylogenetic trees. In this section, we consider the relationship between the rigid hybrid number of two phylogenetic trees and three closely related hybrid numbers (the temporal, weak, and beaded hybrid numbers), in particular, showing that they can all be different from one another. From a biological viewpoint, the results that we present in this section are important as they show that in principle quite different estimates can arise for the number of reticulation events required to accommodate two phylogenetic trees depending on the model being used to embed the two trees in a phylogenetic network.

### The temporal hybrid number

For two phylogenetic trees ${{\mathscr {T}}}$ and ${{\mathscr {T}}}'$ on *X* that can be displayed by some temporal tree-child network, the *temporal hybrid number* of ${{\mathscr {T}}}$ and ${{\mathscr {T}}}'$ (Humphries et al. 2013a) is defined as

$$\begin{aligned}&h_t({{\mathscr {T}}},{{\mathscr {T}}}') =\min \{h({{\mathscr {N}}}) \,:\,\\&\quad {{\mathscr {N}}} \text{ is } \text{ a } \text{ temporal } \text{ tree-child } \text{ network } \text{ that } \text{ displays } {{\mathscr {T}}} \text{ and } {{\mathscr {T}}}'\}. \end{aligned}$$

Note that in case this number exists, the temporal hybrid number for the two phylogenetic trees is not necessarily equal to their hybrid number Humphries et al. (2013a, Figure 1; also p. 1889).

Now, given two phylogenetic trees ${{\mathscr {T}}}$ and ${{\mathscr {T}}}'$ on *X*, Theorem 2 implies that the temporal hybrid number $h_t({{\mathscr {T}}},{{\mathscr {T}}}')$ of ${{\mathscr {T}}}$ and ${{\mathscr {T}}}'$ exists if and only if the rigid hybrid number $h_r({{\mathscr {T}}},{{\mathscr {T}}}')$ exists. Clearly, if these numbers both exist, then $h_r({{\mathscr {T}}},{{\mathscr {T}}}') \le h_t({{\mathscr {T}}},{{\mathscr {T}}}')$. In this section, we show that the difference $h_t({{\mathscr {T}}},{{\mathscr {T}}}')-h_r({{\mathscr {T}}},{{\mathscr {T}}}')$ can grow at least as a linear function of |*X*|.

**Fig. 7:**
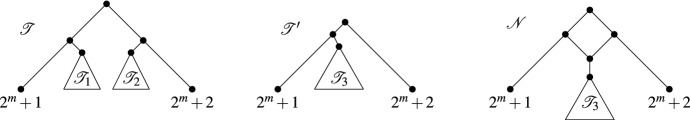
Two phylogenetic trees ${{\mathscr {T}}}$ and ${{\mathscr {T}}}'$ on the set $X=\{1,2,\ldots ,2^m+2\}$ as defined in the text. Both are rigidly displayed by the depicted temporal tree-child network ${{\mathscr {N}}}$ on *X*

**Fig. 8:**
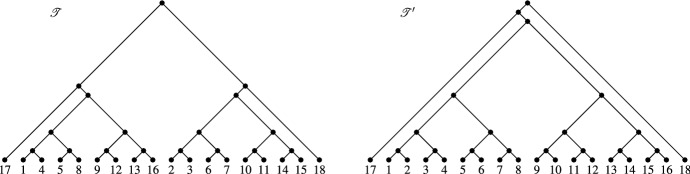
The two phylogenetic trees ${{\mathscr {T}}}$ and ${{\mathscr {T}}}'$ in Fig. 7 for the case $m=4$

To this end, assume that $m \ge 3$. Consider two phylogenetic trees ${{\mathscr {T}}}$ and ${{\mathscr {T}}}'$ with $2^m+2$ leaves, as given in Fig. 7. In that figure, ${{\mathscr {T}}}_1$ and ${{\mathscr {T}}}_2$ are both fully balanced phylogenetic trees with $2^{m-1}$ leaves each and ${{\mathscr {T}}}_3$ is a fully balanced phylogenetic tree with $2^m$ leaves. We next describe the labeling of ${{\mathscr {T}}}$ and ${{\mathscr {T}}}'$. Let $\{{{\mathscr {S}}}'_1,{{\mathscr {S}}}'_2,\ldots ,{{\mathscr {S}}}'_{2^{m-2}}\}$ be the set of all pendant subtrees on four leaves in ${{\mathscr {T}}}_3$. Then, for each $i\in \{1,2,\ldots ,2^{m-2}\}$, we bijectively label the leaves of ${{\mathscr {S}}}'_i$ with $\{4i-3,4i-2,41-1,4i\}$. Turning to the subtrees ${{\mathscr {T}}}_1$ and ${{\mathscr {T}}}_2$ of ${{\mathscr {T}}}$, let $\{{{\mathscr {S}}}_1,{{\mathscr {S}}}_3,\ldots ,{{\mathscr {S}}}_{2^{m-2}-1}\}$ be the set of all pendant subtrees on four leaves in ${{\mathscr {T}}}_1$ and, similarly, let $\{{{\mathscr {S}}}_2,{{\mathscr {S}}}_4,\ldots ,{{\mathscr {S}}}_{2^{m-2}}\}$ be the set of all pendant subtrees on four leaves in ${{\mathscr {T}}}_2$. Then, for each

$$\begin{aligned} i\in \{1,3,\ldots ,2^{m-2}-1\}, \end{aligned}$$

bijectively label the eight leaves of ${{\mathscr {S}}}_i$ and ${{\mathscr {S}}}_{i+1}$ with $\{4i-3,4i-2,\ldots ,4i+4\}$ such that ${{\mathscr {S}}}_i$ has cherries $\{4i-3,{4}i\}$ and $\{4i+1,4i+4\}$ and ${{\mathscr {S}}}_{i+1}$ has cherries $\{4i-2,4i-1\}$ and $\{4i+2,4i+3\}$. For $m=4$, the leaf labeling of ${{\mathscr {T}}}$ and ${{\mathscr {T}}}'$ is illustrated in Fig. 8.

#### Theorem 4

For the two phylogenetic trees ${{\mathscr {T}}}$ and ${{\mathscr {T}}}'$ on $X=\{1,\ldots ,2^m+2\}$, $m\ge 3$, pictured in Fig. 7, we have $h_t({{\mathscr {T}}},{{\mathscr {T}}}')-h_r({{\mathscr {T}}},{{\mathscr {T}}}') \ge \frac{|X|}{4} - 3$.

#### Proof

First note that $h_r({{\mathscr {T}}},{{\mathscr {T}}}')=1$, as ${{\mathscr {T}}}$ and ${{\mathscr {T}}}'$ are rigidly displayed by the temporal tree-child network ${{\mathscr {N}}}$ pictured in Fig. 7.

We now show that $h_t({{\mathscr {T}}},{{\mathscr {T}}}')\ge 2^{m-2}-1$ from which the theorem follows. First note that, by Theorem 2, there must exist a temporal tree-child network that displays both ${{\mathscr {T}}}$ and ${{\mathscr {T}}}'$. By Humphries et al. (2013b, Theorem 3.3), it follows that $h_t({{\mathscr {T}}},{{\mathscr {T}}}')$ is equal to the number of components in a maximum temporal agreement forest for ${{\mathscr {T}}}$ and ${{\mathscr {T}}}'$ minus 1, where such a forest is defined as follows. All phylogenetic trees considered in the definition are “planted" by adding a new root plus an edge to their roots, and trees with one leaf are also allowed. Let $k=h_t({{\mathscr {T}}},{{\mathscr {T}}}')$. Then a collection $\{{{\mathscr {T}}}_0,\ldots ,{{\mathscr {T}}}_k\}$ of $k+1$ planted trees is a maximum temporal agreement forest for (planted versions of) ${{\mathscr {T}}}$ and ${{\mathscr {T}}}'$ if the following three properties hold, where $X_i=L({{\mathscr {T}}}_i)$, $0\le i\le k$.

1. The set $Z=\{X_0, \dots X_k\}$ is a partition of *X*.
2. For all $$0 \le i \le k$$, $${{\mathscr {T}}}_i \cong {{\mathscr {T}}}|_{X_i} \cong {{\mathscr {T}}}'|_{X_i}$$.
3. Denoting for $0\le i\le k$ the root of ${{\mathscr {T}}}_i$ by $\rho _i$, there exist injective maps
  $$\begin{aligned} \chi : \{\rho _0,\rho _1,\ldots ,\rho _k\} \rightarrow V({{\mathscr {T}}}) \text { and } \chi ': \{\rho _0,\rho _1,\ldots ,\rho _k\} \rightarrow V({{\mathscr {T}}}') \end{aligned}$$
  such that any two trees in $\{{{\mathscr {T}}}(X_i\cup \{\chi (\rho _i)\}):0\le i\le k\}$ and $\{{{\mathscr {T}}}'(X_i\cup \{\chi '(\rho _i)\}):0\le i\le k\}$ are edge-disjoint rooted subtrees of ${{\mathscr {T}}}$ and ${{\mathscr {T}}}'$, respectively.

We now claim that for every subset $\{4i-3,\ldots ,4i\}$ of *X* with $1 \le i \le 2^{m-2}$, at least one of the sets $\{4i-3\}$, $\{4i-2\}$, and $\{4i-3, 4i-2\}$ must be contained in *Z*. This implies that $k+1 \ge 2^{m-2}$ from which the theorem immediately follows. For simplicity, we prove the claim for $i=1$. Specifically, we show that, for the subset $\{1,2,3,4\}$ of *X*, at least one of $\{1\}$, $\{2\}$, and $\{1,2\}$ is an element of *Z*. The argument for the remaining cases $2 \le i \le 2^{m-2}$ is similar.

Assume that $\{1,2\}$ is not an element of *Z*. Since *Z* is a partition of *X*, one of the following three cases must hold:

- $\{1\}$ or $\{2\}$ is an element in *Z*,
- $\{1,2\}\cup Y_1$ is an element in *Z*, or
- $\{1\}\cup Y_2$ and $\{2\}\cup Y_3$ are elements in *Z*,

where $Y_1$, $Y_2$, and $Y_3$ are subsets of $X-\{1,2\}$. Now, observe that Case (c) contradicts Property (P3); particularly $\{1\}\cup Y_2$ and $\{2\}\cup Y_3$ are not edge-disjoint in ${{\mathscr {T}}}'$ because ${{\mathscr {T}}}'$ has a cherry $\{1,2\}$. Moreover, Case (b) contradicts Property (P2) because ${{\mathscr {T}}}|_{\{1,2,y\}}$ and ${{\mathscr {T}}}'|_{\{1,2,y\}}$ are non isomorphic, where *y* is an element in $Y_1$. Hence, Case (a) must hold. Thus, one of $\{1\}$, $\{2\}$, and $\{1,2\}$ is an element of *Z*. $\square$

### The weak and beaded hybrid numbers

We now consider the relationship between the rigid, temporal and so-called weak and beaded hybrid numbers. First, given two phylogenetic trees ${{\mathscr {T}}}$ and ${{\mathscr {T}}}'$ on *X*, we define the *weak hybrid number* $h_{wd}({{\mathscr {T}}},{{\mathscr {T}}}')$ of ${{\mathscr {T}}}$ and ${{\mathscr {T}}}'$ as

$$\begin{aligned}&h_{wd}({{\mathscr {T}}},{{\mathscr {T}}}')\\= & {} \min \{h({{\mathscr {N}}}): {{\mathscr {N}}}\text { is a phylogenetic network that weakly displays }{{\mathscr {T}}}\text { and }{{\mathscr {T}}}'\}. \end{aligned}$$

Note that for any two phylogenetic trees ${{\mathscr {T}}}$ and ${{\mathscr {T}}}'$ there always exists a phylogenetic network that displays ${{\mathscr {T}}}$ and ${{\mathscr {T}}}'$. Hence, $h_{wd}({{\mathscr {T}}},{{\mathscr {T}}}')$ is well-defined. The weak hybrid number has been considered implicitly in The Parental Tree Network Problem (Zhu et al. 2016, Definition 5).

Next, recall that a *beaded tree* ${{\mathscr {B}}}$ on *X* is a phylogenetic network on *X* in which parallel edges are allowed, and in which each reticulation vertex *v* has a unique parent *u* such that there are two parallel edges from *u* to *v* (van Iersel et al. 2018, Definition 7). In van Iersel et al. (2018, Definition 6) the concept of a weak embedding of a multi-labeled tree into a beaded tree is defined, which is closely related to our definition of a display map when restricted to phylogenetic trees.^1^ For two phylogenetic trees ${{\mathscr {T}}}$ and ${{\mathscr {T}}}'$ on *X*, we define the *beaded hybrid number* $h_b({{\mathscr {T}}},{{\mathscr {T}}}')$ for ${{\mathscr {T}}}$ and ${{\mathscr {T}}}'$ to be

$$\begin{aligned} h_b({{\mathscr {T}}},{{\mathscr {T}}}')= & {} \min \{h({{\mathscr {B}}}): {{\mathscr {B}}}\text { is a beaded tree such that there exist weak }\\&\text { embeddings of } {{\mathscr {T}}}\text { and }{{\mathscr {T}}}' \text { into } {{\mathscr {B}}}\}. \end{aligned}$$

**Fig. 9:**
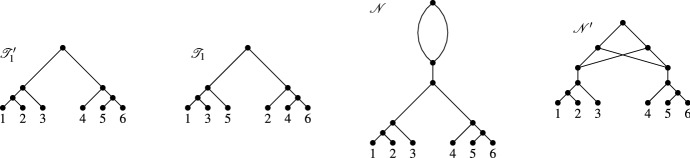
Two phylogenetic trees ${{\mathscr {T}}}_1$ and ${{\mathscr {T}}}'_1$ on $X=\{1,\ldots , 6\}$ whose beaded hybrid number $h_b({{\mathscr {T}}}_1,{{\mathscr {T}}}'_1)$ is one (${{\mathscr {N}}}$ is a beaded tree into which ${{\mathscr {T}}}_1$ and ${{\mathscr {T}}}'_1$ can be weakly embedded), and whose weak hybrid number $h_{wd}({{\mathscr {T}}}_1,{{\mathscr {T}}}'_1)$ is two (the network ${{\mathscr {N}}}'$ weakly displays both trees)

In the following result we summarize the relationship between the various hybrid numbers that we have considered:

#### Theorem 5

Let ${{\mathscr {T}}}$ and ${{\mathscr {T}}}'$ denote two phylogenetic trees on *X*. Then

$$\begin{aligned} h_b({{\mathscr {T}}},{{\mathscr {T}}}') \le h_{wd}({{\mathscr {T}}},{{\mathscr {T}}}'). \end{aligned}$$

Moreover, if the rigid (or equivalently temporal) hybrid number for ${{\mathscr {T}}}$ and ${{\mathscr {T}}}'$ exists, then

$$\begin{aligned} h_b({{\mathscr {T}}},{{\mathscr {T}}}') \le h_{wd}({{\mathscr {T}}},{{\mathscr {T}}}') \le h_r({{\mathscr {T}}},{{\mathscr {T}}}') \le h_t({{\mathscr {T}}},{{\mathscr {T}}}'), \end{aligned}$$

and in this case there exist trees ${{\mathscr {T}}}$ and ${{\mathscr {T}}}'$ such that every inequality is strict.

#### Proof

To see that the first statement holds note that (van Iersel et al. 2018, Lemma 9) implies that any phylogenetic network ${{\mathscr {N}}}$ on *X* that weakly displays two phylogenetic trees ${{\mathscr {T}}}$ and ${{\mathscr {T}}}'$ on *X* can be transformed into a beaded tree ${{\mathscr {B}}}$ on *X* such that there exist weak embeddings of ${{\mathscr {T}}}$ and ${{\mathscr {T}}}'$ into ${{\mathscr {B}}}$ for which $|Ret({{\mathscr {N}}})|=|Ret({{\mathscr {B}}})|$ (so in particular $h_b({{\mathscr {T}}},{{\mathscr {T}}}')$ exists for any pair of phylogenetic trees ${{\mathscr {T}}}$ and ${{\mathscr {T}}}'$). Hence, $h_b({{\mathscr {T}}},{{\mathscr {T}}}') \le h_{wd}({{\mathscr {T}}},{{\mathscr {T}}}')$.

Now, consider the second statement in the theorem. Suppose that $h_r({{\mathscr {T}}},{{\mathscr {T}}}')$ exists (or equivalently, that $h_t({{\mathscr {T}}},{{\mathscr {T}}}')$ exists). By the first statement in the theorem $h_b({{\mathscr {T}}},{{\mathscr {T}}}') \le h_{wd}({{\mathscr {T}}},{{\mathscr {T}}}')$, $h_{wd}({{\mathscr {T}}},{{\mathscr {T}}}') \le h_r({{\mathscr {T}}},{{\mathscr {T}}}')$ clearly also holds, and as remarked in the previous section, $h_r({{\mathscr {T}}},{{\mathscr {T}}}') \le h_t({{\mathscr {T}}},{{\mathscr {T}}}')$. Hence, all of the inequalities in the second statement hold.

We now show that there exist two phylogenetic trees ${{\mathscr {T}}}$ and ${{\mathscr {T}}}'$ such that every inequality is strict. Consider the trees ${{\mathscr {T}}}_1$ and ${{\mathscr {T}}}'_1$ on $X=\{1,\ldots , 6\}$ depicted in Fig. 9. Note that $h_b({{\mathscr {T}}}_1,{{\mathscr {T}}}'_1)=1$ since ${{\mathscr {T}}}_1$ and ${{\mathscr {T}}}'_1$ are not isomorphic, and there exist weak embeddings for ${{\mathscr {T}}}_1$ and ${{\mathscr {T}}}'_1$ into the pictured beaded tree ${{\mathscr {N}}}$, respectively. Moreover, by considering fork-picking sequences it is straight-forward to check that $h_r({{\mathscr {T}}}_1,{{\mathscr {T}}}'_1) = 2$, and by considering cherry picking sequences and using Humphries et al. (2013a, Theorem 2), it is straight-forward to check that $h_t({{\mathscr {T}}}_1,{{\mathscr {T}}}'_1)=4$. Hence, as ${{\mathscr {T}}}_1$ and ${{\mathscr {T}}}'_1$ are not isomorphic, $1 \le h_{wd}({{\mathscr {T}}}_1,{{\mathscr {T}}}'_1) \le h_r({{\mathscr {T}}}_1,{{\mathscr {T}}}'_1) = 2$. In the Appendix we show that $h_{wd}({{\mathscr {T}}}_1,{{\mathscr {T}}}'_1) \ne 1$, and so it follows that $h_{wd}({{\mathscr {T}}}_1,{{\mathscr {T}}}'_1) = 2$.

**Fig. 10:**
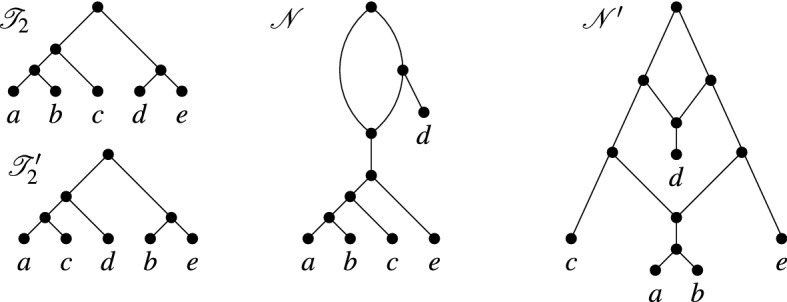
Two phylogenetic trees ${{\mathscr {T}}}_2$ and ${{\mathscr {T}}}'_2$ on $Y=\{a,\ldots , e\}$ whose weak hybrid number is $h_{wd}({{\mathscr {T}}}_2,{{\mathscr {T}}}'_2)=1$ (see ${{\mathscr {N}}}$ for a phylogenetic network that weakly displays both trees) and whose rigid hybrid number is $h_r({{\mathscr {T}}}_2,{{\mathscr {T}}}'_2)=2$ (see ${{\mathscr {N}}}'$ for a temporal tree-child network which rigidly displays ${{\mathscr {T}}}_2$ and ${{\mathscr {T}}}'_2$)

Next consider the two phylogenetic trees ${{\mathscr {T}}}_2$ and ${{\mathscr {T}}}'_2$ on the set $Y= \{a,b,c,d,e\}$ in Fig. 10 in the Appendix. Then using similar arguments to those used to determine the various hybrid numbers for the trees in Fig. 9, it is straight-forward to check that $h_b({{\mathscr {T}}}_2,{{\mathscr {T}}}'_2) = h_{wd}({{\mathscr {T}}}_2,{{\mathscr {T}}}'_2)=1$, $h_r({{\mathscr {T}}}_2,{{\mathscr {T}}}'_2)=2$, and $h_t({{\mathscr {T}}}_2,{{\mathscr {T}}}'_2)=3$.

Now, to complete the proof, consider the phylogenetic trees ${{\mathscr {T}}}$ and ${{\mathscr {T}}}'$ on the set $X \cup Y$ which are constructed as follows: For ${{\mathscr {T}}}$ (respectively, ${{\mathscr {T}}}'$) take a new root vertex and join this vertex via two edges to the roots of the trees ${{\mathscr {T}}}_1$ and ${{\mathscr {T}}}_2$ (respectively, ${{\mathscr {T}}}_1'$ and ${{\mathscr {T}}}_2'$). Then it is straightforward to check that $h_*({{\mathscr {T}}},{{\mathscr {T}}}')=h_*({{\mathscr {T}}}_1,{{\mathscr {T}}}'_1)+h_*({{\mathscr {T}}}_2,{{\mathscr {T}}}'_2)$ for $h_*=h_b,h_{wd},h_r,h_t$. It follows that

$$\begin{aligned} h_b({{\mathscr {T}}},{{\mathscr {T}}}') = 2< h_{wd}({{\mathscr {T}}}_2,{{\mathscr {T}}}'_2)=3< h_r({{\mathscr {T}}}_2,{{\mathscr {T}}}'_2) =4 < h_r({{\mathscr {T}}}_2,{{\mathscr {T}}}'_2)=7. \end{aligned}$$

$$\square $$

## Discussion

Motivated by the fact that in some evolutionary scenarios the notion of a network displaying two phylogenetic trees might be too restrictive, we have introduced and studied the concept of a network rigidly displaying a pair of phylogenetic trees. We have characterized when two trees can be rigidly displayed by a temporal tree-child network and, provided this is possible, have shown that their rigid hybrid number is given by a minimum weight fork-picking sequence for them. In addition, we have shown that the rigid hybrid number is different from the closely related temporal, weak, and beaded hybrid numbers.

There remain several open problems. First, it is well-known that the hybrid number is closely related to the size of a maximum agreement forest for two phylogenetic trees (Baroni et al. 2005a). It would therefore be of interest to know if there is some analogue of a maximum agreement forest for rigidly displaying two trees. Results in Humphries et al. (2013b), including the one mentioned above, concerning temporal agreement forests for two phylogenetic trees to be displayed by temporal tree-child networks could be useful for studying this question. In addition, it could be interesting to define and study rigid hybrid numbers for three or more phylogenetic trees. For example, we could try to understand *r*-rigidly displaying, where *r* is the maximum number of edges that come together at each reticulation vertex (note that in this paper we have investigated the concept of *r*-rigidly displaying for $r=3$). Recently, there has been work on understanding the hybrid number for arbitrary sets of trees (Linz and Semple 2019) which might be relevant.

More generally, several questions remain concerning the notion of weakly displaying. For example, it would be interesting to know how large the difference can potentially be between the hybrid number and the weak hybrid number for a collection of phylogenetic trees. As this appears to be a difficult problem, it might be worth first restricting to the case of understanding the “weak temporal tree-child hybrid number"; how different can this number be from the rigid hybrid number, and can we decide when a set of trees is weakly displayed by a temporal tree-child network? To answer these questions it could be worth first trying to decide whether or not two phylogenetic trees are rigidly displayed by some temporal tree-child network if and only if they are weakly displayed by some temporal tree-child network. In another direction, it would be interesting to understand how the weak- and rigid hybrid numbers behave for other classes of networks besides temporal tree-child networks.

Finally, an important open problem is to develop practical algorithms to compute networks with minimum rigid and/or weak hybrid numbers for two (or more) phylogenetic trees. The complexity of computing the weak and rigid hybrid numbers is unknown (see also Zhu et al. 2016, p.278), but we suspect these will probably be NP-hard in general. We base this suspicion in part on the fact that it is known that computing the minimum hybrid and temporal hybrid number for two trees is NP-hard (Bordewich and Semple 2007b; Humphries et al. 2013a). However, by gaining a better understanding of fork-picking sequences it may be possible to at least develop fixed parameter tractable algorithms to construct optimal networks, an approach that has already proven successful for hybrid and temporal hybrid numbers (Bordewich and Semple 2007a; Humphries et al. 2013a).

## Appendix

In this appendix we prove that $h_{wd}({{\mathscr {T}}}_1,{{\mathscr {T}}}'_1) \ne 1$ for the two phylogenetic trees ${{\mathscr {T}}}_1$ and ${{\mathscr {T}}}'_1$ on $X=\{1,\ldots , 6\}$ pictured in Fig. 9. To simplify notation, we put ${{\mathscr {T}}}= {{\mathscr {T}}}_1$ and ${{\mathscr {T}}}' = {{\mathscr {T}}}'_1$.

Suppose to the contrary that $h_{wd}({{\mathscr {T}}},{{\mathscr {T}}}') =1$. Then there exists a phylogenetic network ${{\mathscr {N}}}^*$ that weakly displays ${{\mathscr {T}}}$ and ${{\mathscr {T}}}'$ such that $h({{\mathscr {N}}}^*)=1$. Let *v* be the unique vertex in $Ret({{\mathscr {N}}}^*)$. Let $u\in V({{\mathscr {N}}}^*)-\{\rho _{{{\mathscr {N}}}^*}\}$ be a parent of *v*. Note that since ${{\mathscr {N}}}^*$ has no parallel edges, *u* must exist. Also note that *u* must be a tree vertex of ${{\mathscr {N}}}^*$ as *v* is the sole reticulation vertex of ${{\mathscr {N}}}^*$. Finally, note that the other child of *u* cannot be *v* as ${{\mathscr {N}}}^*$ does not contain parallel edges. Denoting that child of *u* by *x*, we next claim that *x* must be a leaf of ${{\mathscr {N}}}^*$.

To see the claim, assume for contradiction that *x* is not a leaf. Let ${{\mathscr {T}}}^*$ denote the subtree of ${{\mathscr {N}}}^*$ rooted at *x*. Let $x'$ be a leaf of ${{\mathscr {T}}}^*$ and, thus, of ${{\mathscr {N}}}^*$. Then since ${{\mathscr {N}}}^*$ weakly displays ${{\mathscr {T}}}$ via a map $\psi$ say, and *v* is the sole reticulation vertex of ${{\mathscr {N}}}^*$ we obtain $\gamma _{\psi }(u)\ge 1$. Similarly, as ${{\mathscr {N}}}^*$ weakly displays ${{\mathscr {T}}}'$ via a map $\psi '$ say, $\gamma _{\psi '}(u)\ge 1$ must hold. Thus, $\gamma _{\psi ,\psi '}(u)\ge 2$. Since Lemma 2(i) implies that $\gamma _{\psi ,\psi '}(u)\le 2$ as *v* is the sole reticulation vertex of ${{\mathscr {N}}}^*$, it follows that $\gamma _{\psi ,\psi '}(u)=2$. Hence, by Lemma 2(ii), ${{\mathscr {T}}}^*$ is also a pendant subtree of ${{\mathscr {T}}}$ and of ${{\mathscr {T}}}'$; a contradiction as ${{\mathscr {T}}}$ and ${{\mathscr {T}}}'$ are the two trees depicted in Fig. 9. Thus, *x* is a leaf of ${{\mathscr {N}}}^*$, as claimed.

Since every element in *X* is contained in a cherry of ${{\mathscr {T}}}$ or ${{\mathscr {T}}}'$, we may choose some $y \in X-\{x\}$ such that $\{x,y\}$ is a cherry in ${{\mathscr {T}}}$ or ${{\mathscr {T}}}'$. Without loss of generality, assume that ${{\mathscr {T}}}$ is that tree. Since the only two cherries of ${{\mathscr {T}}}$ are $\{1,3\}$ and $\{4,6\}$ we may assume without loss of generality that $\{x,y\}=\{1,3\}$. Let *m* denote the parent of *x* and *y* in ${{\mathscr {T}}}$.

Let $w\in V({{\mathscr {N}}}^*)$ be the parent of *u* which must exist as $u\not =\rho _{{{\mathscr {N}}}^*}$. Then $w\not =\rho _{{{\mathscr {N}}}^*}$ as otherwise the fact that $\{x,y\}$ is a cherry of ${{\mathscr {T}}}$ but not of ${{\mathscr {T}}}'$ implies that *y* is below *v*. But then ${{\mathscr {T}}}$ is not weakly displayed by ${{\mathscr {N}}}^*$ because $(\rho _{{{\mathscr {N}}}^*},u)$ is an edge of ${{\mathscr {N}}}^*$ and $(\rho _{{{\mathscr {T}}}}, m)$ is not an edge in ${{\mathscr {T}}}$; a contradiction.

We next claim that (*w*, *v*) cannot be an edge in ${{\mathscr {N}}}^*$. To see this, assume for contradiction that (*w*, *v*) is an edge in ${{\mathscr {N}}}^*$. Then since ${{\mathscr {T}}}$ is weakly displayed by ${{\mathscr {N}}}^*$ and *x* is contained in a cherry of ${{\mathscr {T}}}$ but not of ${{\mathscr {T}}}'$ it follows that *y* must be a leaf of ${{\mathscr {N}}}$ below *v*. If there existed another leaf of ${{\mathscr {N}}}^*$ below *v* then that leaf would have to be leaf 5. Since $\{5,6\}$ is a cherry of ${{\mathscr {T}}}'$ and ${{\mathscr {T}}}'$ is weakly displayed by ${{\mathscr {N}}}^*$ it follows that that cherry must also be below *v*; a contradiction as ${{\mathscr {T}}}$ is the phylogenetic tree depicted in Fig. 9. Thus, *y* is in fact the sole leaf of ${{\mathscr {N}}}^*$ below *v*. But then $\{x,y\}$ must also be a cherry of ${{\mathscr {T}}}'$; a contradiction since ${{\mathscr {T}}}$ and ${{\mathscr {T}}}'$ are the phylogenetic trees depicted in Fig. 9. Thus, (*w*, *v*) cannot be an edge in ${{\mathscr {N}}}^*$, as claimed. Hence, the other child of *w*, call it *z*, is either a leaf of ${{\mathscr {N}}}^*$ or the root of a pendant subtree of ${{\mathscr {N}}}^*$.

Note first that arguments similar to the case of *x* imply that *z* must be a leaf of ${{\mathscr {N}}}^*$. Let $p\in V({{\mathscr {N}}}^*)$ denote the parent of *w*. We next distinguish between the cases that $z=y$ and $z\not =y$.

If $z=y$ then $p\not =\rho _{{{\mathscr {N}}}^*}$. To see this, assume for contradiction that $p=\rho _{{{\mathscr {N}}}^*}$. Then $(\rho _{{{\mathscr {N}}}^*},w)$ is an edge in ${{\mathscr {N}}}^*$. Since $\{x,y\}$ is a cherry of ${{\mathscr {T}}}$ and the parent *m* of *x* and *y* is not adjacent with $\rho _{{{\mathscr {T}}}}$ it follows that ${{\mathscr {T}}}$ is not weakly displayed by ${{\mathscr {N}}}^*$; a contradiction. Thus, $p\not =\rho _{{{\mathscr {N}}}}$, as required.

We next claim that (*p*, *v*) cannot be an edge of ${{\mathscr {N}}}^*$. Assume for contradiction that (*p*, *v*) is an edge of ${{\mathscr {N}}}^*$. Then since $\{x,y\}$ is a cherry of ${{\mathscr {T}}}$ and ${{\mathscr {T}}}$ is weakly displayed by ${{\mathscr {N}}}^*$, similar arguments as before imply that leaf 5 must be the sole leaf of ${{\mathscr {N}}}^*$ below *v* and that the unique directed path from $\rho _{{{\mathscr {N}}}^*}$ to leaf 2 does not contain *w*. Since ${{\mathscr {T}}}'$ is one of the two phylogenetic trees depicted in Fig. 9 it follows that ${{\mathscr {T}}}'$ is not weakly displayed by ${{\mathscr {N}}}^*$; a contradiction. Thus, (*p*, *v*) cannot be an edge of ${{\mathscr {N}}}^*$ either.

Let *q* denote the other child of *p*. Then similar arguments as in the case of *z* imply that *q* must also be a leaf of ${{\mathscr {N}}}^*$. Hence, the phylogenetic tree ${\mathscr {R}}$ on $\{q,x,y\}$ with cherry $\{x,y\}$ is a pendant subtree of ${{\mathscr {T}}}$. Since *p* is a tree vertex of ${{\mathscr {N}}}^*$, Lemma 2(ii) implies that ${\mathscr {R}}$ is a pendant subtree of ${{\mathscr {T}}}$ and ${{\mathscr {T}}}'$; a contradiction in view of Fig. 9. Hence, $h_{wd}({{\mathscr {T}}},{{\mathscr {T}}}') \ne 1$ in case $z=y$.

Assume for the remainder that $z\ne y$. Then *y* is a descendant of *v* in ${{\mathscr {N}}}^*$. Since *z* is a leaf of ${{\mathscr {N}}}$, it follows that the phylogenetic tree on $\{x,y,z\}$ with cherry $\{x,y\}$ is a pendant subtree of ${{\mathscr {T}}}$. Since $\{x,y\}= \{1,3\}$ we must have $z=5$. But then $x=6$ as $\{5,6\}$ is a cherry of ${{\mathscr {T}}}'$ and ${{\mathscr {T}}}'$ is weakly displayed by ${{\mathscr {N}}}^*$; a final contradiction. Hence, $h_{wd}({{\mathscr {T}}},{{\mathscr {T}}}') \ne 1$ and, so, Theorem 5 follows.

## Footnotes

1 It differs from our definition of a display map since it allows edges to be mapped to paths of length 0.
